# Genomic insights into within-farm persistence and global phylogenetic relatedness of Shiga toxin-producing *Escherichia coli* O26 in a dairy cattle farm in the UK

**DOI:** 10.3389/fmicb.2026.1786881

**Published:** 2026-04-09

**Authors:** Alannah S. Deeney, Sanjukta Raj Kumari, Susan M. Withenshaw, Harriet L. Bare, Nicholas A. Duggett, Miranda Kirchner, Richard P. Smith, Robert H. Davies, John D. Rodgers, Muna F. Anjum

**Affiliations:** Animal and Plant Health Agency, Addlestone, Surrey, United Kingdom

**Keywords:** cattle, epidemiology, locus of enterocyte effacement, O26, sequence type 21, Shiga toxin-producing *E. coli*, STEC, whole genome sequencing

## Abstract

**Background:**

Shiga toxin-producing *Escherichia coli* (STEC) O26 is increasingly implicated in human hemolytic uremic syndrome globally. As cattle are an important reservoir, we conducted a year-long pilot study on a dairy farm in England (~200 cattle) to investigate the occurrence, persistence, and genomics of STEC O26.

**Methods:**

Across four visits, 250 samples were collected (fecal pats *n* = 143, boot swabs *n* = 58, environmental *n* = 49). All *E. coli* O26 isolates were confirmed by real-time PCR and characterized using short-read whole-genome sequencing (WGS) for serotype, sequence type (ST), clonal complex (CC), phylogeny, *stx* subtyping, locus of enterocyte effacement pathogenicity island, and virulence genes. A Bayesian logistic regression model evaluated associations between STEC presence and predictors.

**Results:**

A total of 23 *E. coli* O26 isolates were recovered from 250 samples, with 14 STEC O26 carrying *stx1, stx2*, and *eae*, and nine non-STEC O26 were identified. Successful sequencing of a subset of 20 *E. coli* O26 confirmed all as O26:H11, of which, 12 STEC belonged to ST21 harboring *stx1a* and *stx2a*, and eight were non-STEC ST29, both being within CC29. Two non-O26 STEC were also recovered (STEC O130:H53 and STEC O145:H12). SNP analysis demonstrated STEC O26 clones were persisting within the herd; while non-STEC ST29 clones only emerged in visits 3 and 4. Although clustering with human STEC O26 isolates, the study isolates did not show any epidemiological or genetic link to human isolates, as they differed by at least 51 SNPs. We observed clear temporal and environmental variation in STEC O26 detection and Bayesian logistic regression modeling showed significantly reduced odds of STEC detection outdoors.

**Conclusions:**

STEC O26:H11 ST21 was consistently detected on a dairy farm in England over a year. Despite low detection rates, the persistence of STEC O26 in cattle and their environment poses a public health risk due to its low infectious dose. These findings provide insight into STEC occurrence and genomic diversity within a cattle farm, its zoonotic risks, and suggest that multiple factors including temporal and housing condition may influence detection.

## 1 Background

Shiga toxin-producing *Escherichia coli* (STEC) are zoonotic pathogens capable of causing severe human disease. The only published global estimate of STEC infections remains approximately 2.8 million cases of acute illness annually, with the actual burden likely higher due to under-reporting (Majowicz et al., 2014), and advances in diagnosis warrant a global burden estimate. While STEC O157 is the most common serogroup in humans in Great Britain, several other STEC serogroups are emerging, as they are increasingly identified in outbreaks and cases of sporadic infection (FAO and WHO, 2018; Efsa Biohaz Panel et al., 2020). In England in 2018, reports of non-O157 STEC in humans had increased four-fold compared to 2014, mostly attributed to the implementation of molecular methods for identifying *stx* genes (Vishram et al., 2021).

One of the predominant non-O157 STEC serogroups causing human illness in England is O26, manifesting clinical symptoms such as bloody diarrhea, vomiting and hemolytic uremic syndrome (HUS; Vishram et al., 2021; Byrne et al., 2014). From 2014 to 2021, there were 596 cases of STEC O26:H11 isolation from humans in England, with sequence type (ST) 21 being the most common (556 cases), followed by ST29 (35 cases), while other STs occurred singly (5 cases). Among these clinical STEC O26:H11 isolates, the Shiga toxin gene subtype *stx1a* was predominant, but the occurrence of *stx2a* alone or in combination with *stx1a* increased during this period (Rodwell et al., 2023).

A recent case-control study conducted from 2019 to 2020 examined animal and environmental risk factors associated with sporadic STEC infection in England. It identified childcare occupation, international travel, the summer season, and petting zoo visits as factors linked to increased risk of STEC O26 infection (Kintz et al., 2023). In Europe, the three most frequently reported sources of foodborne STEC infection are beef (~40%), followed by dairy (~15%), and vegetables (~13%; FAO and WHO, 2018). Cattle are important reservoirs of STEC O26, shedding the bacteria in their feces, but there have been limited empirical investigations of STEC O26 on cattle farms in England, resulting in a lack of understanding regarding its occurrence and persistence in cattle herds and the wider farm environment (Hoyle et al., 2021; Jenkins et al., 2003; Pearce et al., 2004). Given the rising cases of STEC O26 human infection in recent years, it is crucial to enhance our knowledge to improve risk management strategies for STEC infection in this sector of primary production (Rodwell et al., 2023; EFSA and ECDC, 2021).

With the increasing use of whole genome sequencing (WGS) in prevalence studies or outbreak investigations, researchers are now better equipped to perform serotyping, Shiga toxin subtyping, phylogenetic analysis and determining virulence in the genome of any STEC (Dallman et al., 2015; Efsa Biohaz Panel et al., 2020; Treacy et al., 2019). Shiga toxins are the characteristic virulence determinants of STEC; however, STEC may have other associated virulence genes such as presence of the locus of enterocyte effacement (LEE) that comprises genes required for inducing attachment and effacement lesions in the host, such as the intimin (*eae*) and translocated intimin receptor (*tir*) genes (Stevens and Frankel, 2014). Therefore, to understand the occurrence, persistence, and genomics of STEC O26 at the within-herd level, a longitudinal study was conducted on an English dairy farm, where the presence of STEC O26 had been confirmed from an initial STEC screening. This pilot investigation was conducted over a year to provide initial insights into the dynamics of STEC occurrence, as well as molecular and genomic characterization of isolates present within the herd.

## 2 Materials and methods

### 2.1 Study farm

The privately owned study farm was recruited for longitudinal investigation between June-December 2019, after an initial screening visit of 11 farms in 2018, during which a single farm tested positive for STEC O26. It was a closed dairy farm practicing all-year-round calving, housing different age groups of young heifers (4-weeks-old to >20 months-old) and maintaining an adult milking herd (~200 animals), including ~10% dry cows at any one time. The adults were seasonally pastured from March/April and re-housed in October/November, separating dry cows from milkers (Supplementary Table S1). In winter, milkers were housed in free-standing cubicles, while dry cows were housed separately. Youngstock, older than 6 months, were grazed during the summer, grouped by similar age across several fields, of which some were far from the main farm site (S1; Supplementary Table S2), and were housed in loose, straw-bedded pens on the main farm site in winter. Calves (< 6 months) were kept indoors throughout the year. Calves were weaned from dams within 24 h of birth and individually penned initially. At 3 months of age calves were weaned off milk and grouped (average *n* = 10) in straw-bedded pens.

### 2.2 Sampling visits and sampling

#### 2.2.1 Longitudinal sampling visits (visits 2, 3, and 4)

Following detection of STEC O26 at the screening visit 1 (details in Supplementary material), three further sampling visits were conducted to align with the movement of the adult herd to pasture (June), after the herd had been at pasture for approximately 6 months (October), and when subsequently rehoused (December; Supplementary Table S1).

Samples were collected from animal housing at the main farm site (S1), and from 13 other sites (S2–S14) over the study period, determined by the location of animal groups (Supplementary Table S2). At each follow-up visit, adults in the main milking herd (including dry cows) and youngstock (youngest calves to bulling heifers) were sampled; these two broad age categories comprised several different epidemiological groups based on groups of animals housed or grazed in the same location. A total of 32 fecal samples (22 pooled and 10 individual) were collected from each age category at each visit (*n* = 64). If an epidemiological group was split across multiple enclosures, samples were collected proportionately from all enclosures where feasible. Priority was given to collecting samples from enclosures that were previously sampled and still occupied. The sample size provided 95% confidence of detecting STEC O26 within each age category, based on expected within-herd prevalence of 10% for O157 STEC (Paiba et al., 2003), but was not sufficient to provide robust estimates of prevalence.

#### 2.2.2 Sample types and collection methods

Freshly voided pooled floor feces from animals at pasture, and from individually penned pre-weaned calves, were collected by combining feces from each of five separate fecal droppings (pats; ~2 g/pat; 10 g total), randomly selected across the sampling field or five pens. Fecal matter was collected from two locations from each pat to account for heterogeneous distribution of STEC. Individual fecal samples, collected only from enclosures where pooled fecal samples were also collected, comprising ~10 g each from a single pat, were collected at each visit from at least one enclosure per epidemiological group sampled. All fecal samples were collected using a sterile wooden spatula and added or mixed (pooled) directly into a sterile sample pot and sealed. As each pooled sample comprised feces from five cowpats or a swab from the floor of an indoor enclosure, more animals were represented by the pooled samples than for each of the individual samples, which came from a single animal.

Pooled feces from all other indoor enclosures were collected using boot swabs pre-moistened with tryptone salt diluent (Sterisox^®^, Anson (UK) Ltd.), by walking in a shuffling motion. Each sample comprised a single boot swab, which was placed into a sterile pot. All samples were transported at ambient temperature and stored at 4 °C until further processing within 48 h of collection.

Additionally, 16 environmental samples were collected during each of the longitudinal visits, including swabs, pre-moistened with tryptone salt, from various types of vehicle tire/foot wells, water troughs, indoor pen partitions, indoor cattle walkways, pooled farmyard water, and rodent/wild bird feces using sterile wooden spatulas.

### 2.3 Microbiology

Fecal (10 g) and swab samples were enriched in buffered peptone water (90 mL) at 41.5 °C for 24 h. Immunomagnetic separation (IMS) was then performed on 1 mL enrichment using Dynabeads (ThermoFisher Scientific, Basingstoke, UK). For the initial screening visit, a pooled set of serogroup-specific antibody-coated magnetic beads targeting O26, O103, O111, and O145 was used, whereas for the subsequent longitudinal visits only O26 beads were employed (Kirchner et al., 2019). The IMS eluate was spread (100 μl) onto rhamnose (10 g L^−1^) MacConkey agar (peptone 20 g L^−1^, bile salts 5 g L^−1^, sodium chloride 5 g L^−1^, neutral red 0.075 g L^−1^, agar 12 g L^−1^) supplemented with cefixime 0.05 mg L^−1^ and potassium tellurite 2.5 mg L^−1^, and incubated for 18–24 h at 37 °C. Subsequently, white/gray colonies (up to 5) were subcultured onto CHROMagar ECC (CHROMagar, Paris, France) for molecular investigation. A loopful of growth was picked using a 1 μL loop from each plate, suspended in 200 μL of sterile molecular-grade water, and boiled at 100 °C for 15 min for heat inactivation.

### 2.4 Molecular characterization

Molecular detection of *E. coli* O26 was performed on all heat-inactivated presumptive O26 isolates using a multiplex real-time PCR which also detects other serogroups including O103, O111, O145, O157 and O55 (Perelle et al., 2004), and included a 16S rRNA gene internal control (USDA and FSIS, 2019). These PCRs were done, alongside the real-time multiplex PCR for virulence genes *stx1, stx2*, and *eae* (Perelle et al., 2004; Kagkli et al., 2011). For both PCRs, 2 μl of the heat inactivated bacterial lysate (boilate) served as DNA template.

For WGS, DNA was extracted from the heat inactivated boilate using a MagMAX CORE Nucleic Acid Purification Kit and KingFisher Flex system (ThermoFisher Scientific; Duggett et al., 2020) from all STEC and non-STEC isolates (*n* = 25). Short-read WGS was conducted at the APHA Central Sequencing Unit on an Illumina NextSeq platform with Nextera XT DNA Library Preparation Kit (Illumina, California, USA; Duggett et al., 2018); successful WGS was defined as ≥25x genome coverage based on NC_013361 reference genome length 5,697,240 bp. For three STEC O26 isolates we were unable to meet this quality threshold. The genomes of *E. coli* O26 were assembled using SPAdes (v 3.13.1; Prjibelski et al., 2020) and Unicycler (v 0.5.0; Wick et al., 2017). Multilocus sequencing typing was conducted with SRST2 (v 0.2.0; Inouye et al., 2014) using *E. coli* (Achtman) pubMLST scheme, and clonal complex designation was determined by *E. coli* Achtman pubMLST scheme (Wirth et al., 2006). Virulence and Shiga toxin gene characterization was performed using the APHASeqFinder pipeline (v5.0.0; AbuOun et al., 2021), using the virulence database https://github.com/APHA-AMR-VIR/APHASeqFinder/blob/master/APHASeqFinder_5.0.0/references/virulence/VirulenceFactors_2024_06_18.fna; and Shiga toxin subtyping database https://github.com/APHA-AMR-VIR/APHASeqFinder/blob/master/APHASeqFinder_5.0.0/references/Shiga_toxin_stx_subtyping_db_19052023.fna.

Assembled genomes of publicly available *E. coli* O26 ST21 (*n* = 6,990) and ST29 (*n* = 813) were downloaded from EnteroBase (Zhou et al., 2020) and compared with study isolates using Mashtree (v 1.4.6) with options –genome size 5000000 –sort-order random (Katz et al., 2019); ST21 and ST29 comparisons were performed separately. Using an in-house python script, 150 public isolates closest to the representative study isolates (ST21: V0147; ST29: V0349), were identified from the Mashtree distance matrix. From these, only public isolates with available metadata (sequence read archive number, source, country, and year) were chosen, resulting in 126 ST21 and 108 ST29 for further analysis. Raw-read sequences of both study and public isolates were analyzed separately for ST21 and ST29 using Snippy (v 4.6.0) to generate SNP alignments (Seemann, 2020). *E. coli* O26:H11 ST21 isolate 11368 (NC_013361) served as the reference genome for both analyses (Ogura et al., 2009), as no fully resolved ST29 reference genome was publicly available. The Snippy generated core.full.aln was further processed using SNP-sites (Page et al., 2016) to retain all SNPs irrespective of gaps/N, while filtering out all invariant sites to reduce computational burden. Maximum likelihood phylogenetic trees were then constructed using RAxML-NG (Kozlov et al., 2019) with options –model GTR+G –seed 2 –tree pars{25} –bs-trees 1000. Phylogenetic trees were annotated in R, using ggtree and gheatmap packages (Yu, 2022). Gene presence/absence figures were generated using ggplot2 with geom_tile (Hadley, 2016). Clonality of the O26 study isolates was determined by generating a pairwise SNP-distance matrix from the Snippy core.full.aln, with NC_013361 as the reference, using snp-dists (v 0.8.0; Seemann, 2021). Isolates were categorized as follows based on pairwise SNP-distance: clonal ≤ 10 SNPs, very closely related >10 and ≤ 20 SNPs, and closely related >20 and ≤ 30 SNPs.

### 2.5 Statistical analysis

Chi squared tests were used to identify whether STEC O26 positivity varied across categorical factors such as age group, sample type, season, and sampling location (*P* < 0.05). In contrast, a Bayesian logistic regression model was applied to quantify the strength and direction of association between sample-level predictors and a binary outcome (STEC presence or absence) using the stan_glm function in R (version 4.4.3) with a binomial family and logit link (Goodrich et al., 2025). The predictors included location (Indoor vs. Outdoor), sample type (pooled feces, individual feces, environmental), and visit (Visit1, Visit2, Visit3, Visit4). The model used 4,000 Markov Chain Monte Carlo samples to estimate parameters. Convergence was assessed via Rhat and n_eff, and model fit was evaluated through posterior predictive checks. Predictor effects were interpreted using ORs, 95% credible intervals (CI), and P[Odds Ratio (OR) > 1; Details in Supplementary Table S10, and Supplementary Figures S2–S7].

## 3 Results

### 3.1 Occurrence of STEC and non-STEC across all farm visits

Overall, 14 STEC O26 (5.6%), nine non-STEC O26 (3.6%) isolates, confirmed by PCR, were recovered from 250 fecal and environmental samples across all four visits over the one-year period (Table 1). Additionally, two STEC non-O26s (O145 and O130, both positive for *stx1*) were also recovered, with one identified as O145 by PCR, and the other not assigned a serogroup using our PCR panel. More specifically, STEC O26 was recovered from 11/201 (5.4%) fecal samples and 3/49 (6.1%) environmental samples (Table 1). WGS of all 25 isolates further identified 20 as O26:H11, two as O130:H53 and O145:H12, but three PCR-confirmed O26 isolates failed sequencing and were not characterized further (Table 2).

**Table 1:** Overview of type and number of samples collected, and proportions of Shiga toxin-producing *Escherichia coli* O26 positive samples.

Sample type	Visit 1	Visit 2	Visit 3	Visit 4	Total positive samples
Youngstock pooled feces	0/6	0/16	4/16	0/1	4/39 (10.3%)
Youngstock fecal boot swabs	1/3	0/6	0/6	1/21	2/36 (5.6%)
Total positive youngstock pooled samples	1/9	0/22	4/22	1/22	6/75 (8.0%)
Youngstock individual feces	ND^*^	0/10	1/10	0/10	1/30 (3.3%)
Total positive youngstock fecal samples	1/9 (11.1%)	0/32	5/32 (15.6%)	1/32 (3.1%)	7/105 (6.7%)
Adult pooled feces	ND	3/22	0/22	ND	3/44 (6.8%)
Adult fecal boot swabs	ND	ND	ND	0/22	0/22
Total positive adult pooled samples	ND	3/22	0/22	0/22	3/66 (4.5%)
Adult individual feces	ND	1/10	0/10	0/10	1/30 (3.3%)
Total positive adult fecal samples	ND	4/32 (12.5%)	0/32	0/32	4/96 (4.2%)
**Total positive fecal samples**	1/9 (11.1%)	4/64 (6.3%)	5/64 (7.8%)	1/64 (1.6%)	11/201 (5.5%)
Environmental (youngstock enclosure)	ND	1/10	2/9	0/9	3/28 (10.7%)
Environmental (adult enclosure)	0/1	0/6	0/7	0/7	0/21
**Total positive environmental samples**	0/1	1/16 (6.0%)	2/16 (13.0%)	0/16	3/49 (6.1%)
**Total positive samples**	1/10 (10.0%)	5/80 (6.3%)	7/80 (8.8%)	1/80 (1.3%)	14/250 (5.6%)

^*^ND, Not done.

**Table 2:** Metadata and genetic characteristics of Shiga toxin-producing *Escherichia coli* and serogroup O26 *E. coli* isolated during the dairy farm investigation.

Sl. no.	Strain ID	Visit number	Site	Animal age group	Animal location	Sample type	rt-PCR	WGS		
							Serogroup, Shiga toxin 1, Shiga toxin 2, intimin	Serotype	Genotype *stx1a, stx2a, eae*	MLST
1	V0147	1 (screening)	S1	Youngstock	Indoor	Pooled feces	Positive for O26, *stx1, stx2, eae*	O26:H11	*stx1a, stx2a, eae*	21
2	V0322	2	S2	Adult	Pasture	Individual feces	Positive for O26, *stx1, stx2, eae*	O26:H11	*stx1a, stx2a, eae*	21
3	V0323	2	S2	Adult	Pasture	Pooled feces	Positive for O26, *stx1, stx2, eae*	O26:H11	*stx1a, stx2a, eae*	21
4	V0324	2	S2	Adult	Pasture	Pooled feces	Positive for O26, *stx1, stx2, eae*	O26:H11	*stx1a, stx2a, eae*	21
5	V0326	2	S2	Adult	Pasture	Pooled feces	Positive for O26, *stx1, stx2, eae*	O26:H11	*stx1a, stx2a, eae*	21
6	V0328	2	S5	Youngstock	Pasture	Environmental: water trough	Positive for O26, *stx1, stx2, eae*	O26:H11	*stx1a, stx2a, eae*	21
7	V0338	3	S1	Youngstock	Indoor	Pooled feces	Positive for O26, *stx1, stx2, eae*	O26:H11	*stx1a, stx2a, eae*	21
8	V0339	3	S1	Youngstock	Indoor	Environmental: sick pen soiled bedding boot swab	Positive for O26, *stx1, stx2, eae*	O26:H11	*stx1a, stx2a, eae*	21
9	V0340	3	S8	Youngstock	Pasture	Pooled feces	Positive for O26, *eae*. Negative for *stx1, stx2*	O26:H11	*eae*	29
10	V0342	3	S10	Youngstock	Pasture	Pooled feces	Positive for O26, *stx1, stx2, eae*	O26:H11	*stx1a, stx2a, eae*	21
11	V0343	3	S10	Youngstock	Pasture	Pooled feces	Positive for O26, *stx1, stx2, eae*	O26:H11	*stx1a, stx2a, eae*	21
12	V0344	3	S10	Youngstock	Pasture	Pooled feces	Positive for O26, *stx1, stx2, eae*	O26:H11	*stx1a, stx2a, eae*	21
13	V0345	3	S6	Youngstock	Pasture	Pooled feces	Positive for O26, *eae*. Negative for *stx1*, stx*2*	O26:H11	*eae*	29
14	V0346	4	S1	Youngstock	Indoor	Pooled feces	Positive for O26, *stx1, stx2, eae*	O26:H11	*stx1a, stx2a, eae*	21
15	V0348	4	S1	Youngstock	Indoor	Environmental: walkway swab	Positive for O26, *eae*. Negative for *stx1, stx2*	O26:H11	*eae*	29
16	V0349	4	S14	Youngstock	Indoor	Pooled feces	Positive for O26, *eae*. Negative for *stx1, stx2*	O26:H11	*eae*	29
17	V0350	4	S14	Youngstock	Indoor	Pooled feces	Positive for O26, *eae*. Negative for *stx1, stx2*	O26:H11	*eae*	29
18	V0352	4	S14	Youngstock	Indoor	Individual feces	Positive for O26, *eae*. Negative for *stx1, stx2*	O26:H11	*eae*	29
19	V0353	4	S14	Youngstock	Indoor	Individual feces	Positive for O26, *eae*. Negative for *stx1, stx2*	O26:H11	*eae*	29
20	V0354	4	S1	Youngstock	Indoor	Individual feces	Positive for O26, *eae*. Negative for *stx1, stx2*	O26:H11	*eae*	29
STEC O26 isolates confirmed by rt-PCR (failed WGS)										
21	V0336^*^	3	S1	Youngstock	Indoor	Individual feces	Positive for O26, *stx1, stx2, eae*	ND	ND	ND
22	V0337^*^	3	S1	Youngstock	Indoor	Environmental: Pooled water in scrape-through dunging area of pens	Positive for O26, *stx1, stx2, eae*	ND	ND	ND
23	V0351^*^	4	S14	Youngstock	Indoor	Pooled feces	Positive for O26, *eae*. Negative for *stx1, stx2*	ND	ND	ND
STEC non-O26 (O130:H53 and O145:H12)										
24	V0347	4	S1	Youngstock	Indoor	Pooled feces	Positive for O145, *stx1*. Negative for *stx2, eae*	O145:H12	*stx1a*	101
25	V0355	4	S1	Youngstock	Indoor	Individual feces	Positive *for stx1*. Negative for *stx2, eae*	O130:H53	*stx1a*	297

ND, Not Done.

^*^V0336, V0337, and V0351 were confirmed by real-time PCR (rt-PCR) but failed whole genome sequencing (WGS), and so the H antigen, toxin subtypes and multilocus sequence type (MLST) are not shown in this table.

During the first exploratory winter visit (visit 1), STEC O26 was only detected in one pooled youngstock feces; however, no adults were sampled during this initial visit.

At visit 2, five samples tested positive for STEC O26: four adult feces (4/32, including three pooled (3/22) and one individual (1/10) feces) from milkers at site S2, and one environmental sample (1/16) from the outer surface of a water trough at site S5. None of the youngstock feces were positive (0/32). Overall, occurrence was 6.2% (5/80) among the fecal and environmental samples.

At visit 3, seven samples tested positive for STEC O26. These included five youngstock fecal samples (5/32, including pooled (4/22) and individual (1/10) feces), pooled positives were from 9-month-old animals at S10 (*n* = 3) and < 3-month-old at S1 (*n* = 1), while the individual positive was from a 3 to 4-month-old animal at S1 (*n* = 1). Additionally, two STEC O26-positive environmental samples were from S1, including soiled bedding (an empty sick pen previously housing 6–9-months-old animals) and pooled walkway water. Furthermore, two non-STEC O26 isolates were recovered from youngstock pooled feces at pasture sites S6 and S8. No adult samples were positive. Overall, 7/80 (8.75%) of the combined fecal and environmental samples were STEC O26-positive during the third visit.

During the final visit (visit 4), a single boot swab from 3-month-old youngstock at S1 tested positive for STEC O26. No adult fecal or environmental samples were positive. Seven non-STEC O26 isolates were detected from youngstock samples: three pooled feces, three individual feces, and one environmental walkway swab. Additionally, STEC serogroups O145 and O130 were also detected from pooled feces (< 3-months-old), and individual feces (4–5-month-old), respectively, from S1 on this visit (Table 2).

The Bayesian model showed good fit, with the observed event rate (~5.6%, 14/250) closely aligning with the mean of the posterior predictive distribution (mean PPD = 0.0586, Standard Deviation = 0.0202, 80% CI: 0.032–0.084).

### 3.2 Effect of visit on STEC detection

**Figure 1:**
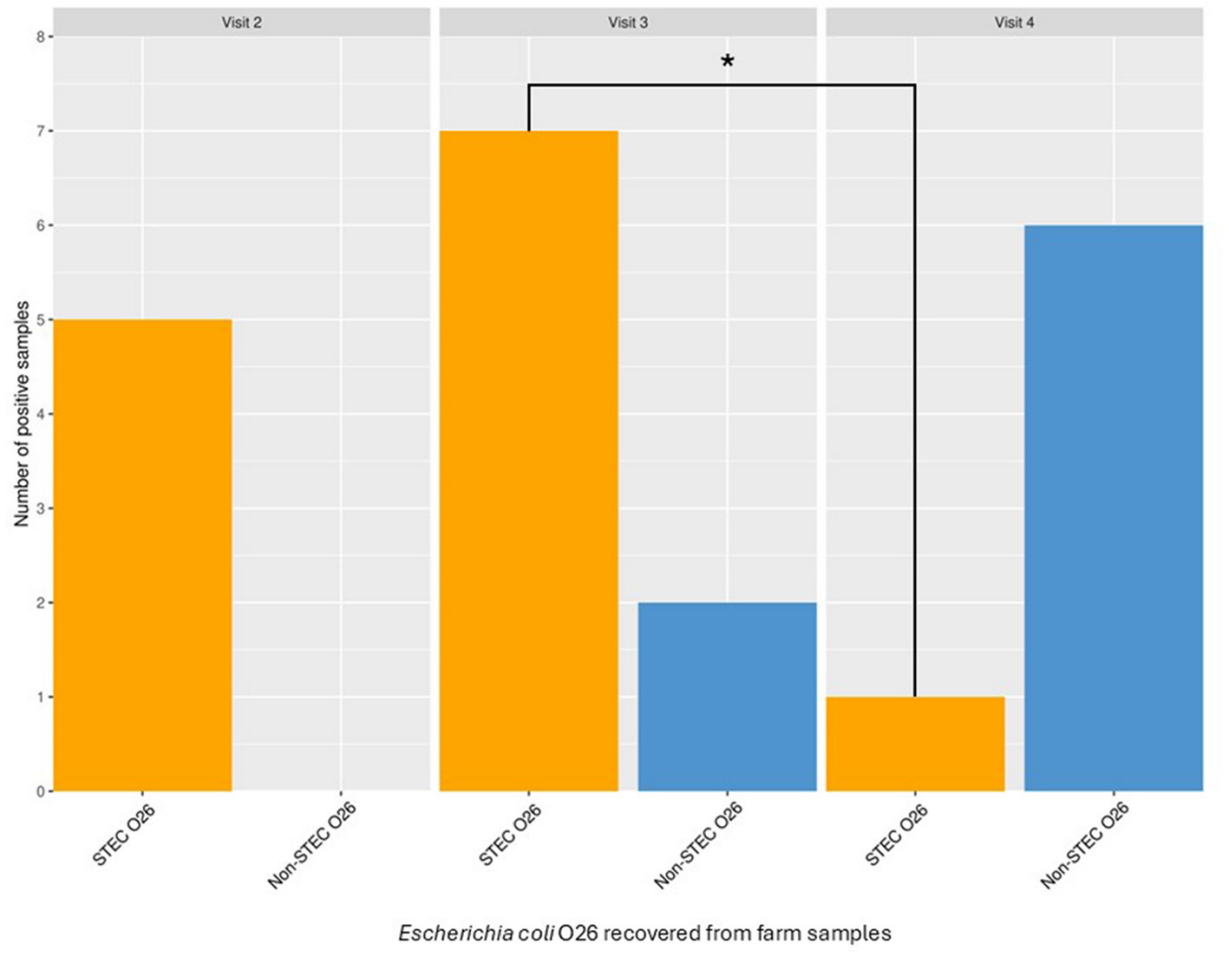
Number of samples positive for STEC O26 and non-STEC O26 during the longitudinal visits 2, 3, and 4 are shown. Association of STEC O26 positivity with visit was tested by Pearson Chi, *P < 0.05.

We observed the highest occurrence of STEC O26 in visit 3 compared to visit 2 and 4; the number of STEC O26-positive samples was significantly associated with visit 3 compared to visit 4 (Chi^2^, *p* = < 0.05; Figure 1). Furthermore, the Bayesian logistic regression model showed a lower OR of detecting STEC O26 at the final visit (Visit 4; OR = 0.108, 95% CI: 0.006–2.337) compared to prior visits (Supplementary Table S10). However, the credible intervals were wide and were not statistically significant, indicating substantial uncertainty about the direction and strength of these effects. Additionally, the STEC O26 positivity rates for both the adults and youngstock ranged between 4.2% and 6.7%. During the longitudinal visits, samples from adults were positive only during visit 2, while the youngstock samples were positive at visits 3 and 4 only.

### 3.3 Effect of pooling samples and location on STEC O26 detection

The recovery of STEC O26 from pooled floor feces (excluding boot swabs) and individual samples were 7/83 (8.4%) and 2/60 (3.3%), respectively (Table 1). Overall, pooled samples demonstrated a relatively higher proportion of STEC O26 positivity compared to individual samples, although this observation was not statistically significant.

The effect of farm sampling location on presence of STEC O26, as estimated by Bayesian logistic regression modeling, indicated that outdoor locations were significantly associated with reduced odds, although the credible intervals were wide indicating a degree of uncertainty around this result [OR = 0.286, 95% CI: 0.074–1.104, P(OR < 1) = 0.965; Supplementary Table S10]. An interesting anecdotal finding was the detection of STEC O26 in a single barn at site S1 at all visits except for visit 2.

Statistical testing comparing STEC O26 positivity and the variables of animal age and sample type (pooled vs. individual), did not yield any significant associations (Supplementary Table S5).

### 3.4 Genome sequence analysis

#### 3.4.1 Genome characterization of STEC and non-STEC O26

**Figure 2:**
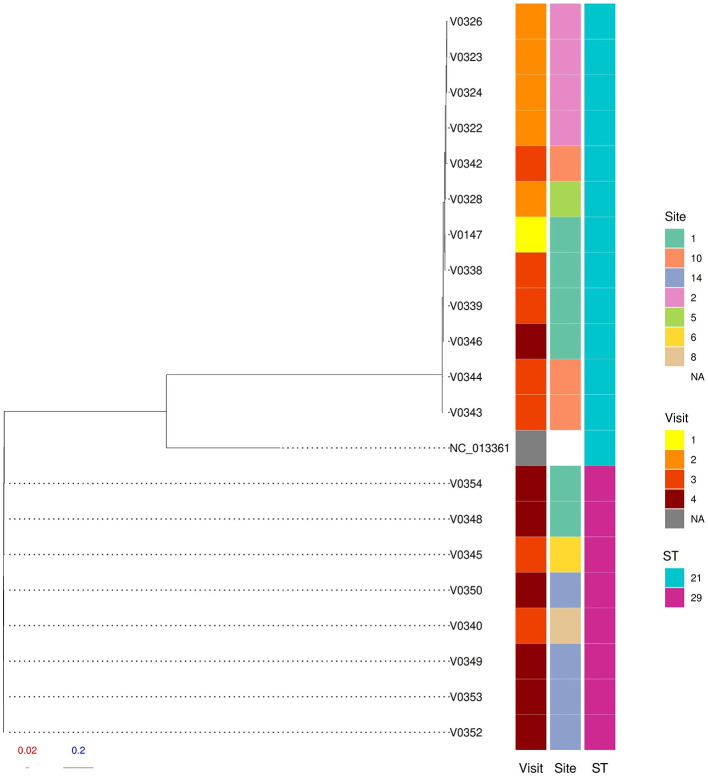
*Escherichia coli* O26 study isolates single nucleotide polymorphism maximum likelihood phylogenetic tree. RAxML-NG tree made from Snippy core.full.aln filtered with SNP-sites. The visit number, site ID, and sequence type (ST) of each isolate are shown in colored columns (left to right). Reference isolate NC_013361 is shown.

**Figure 3:**
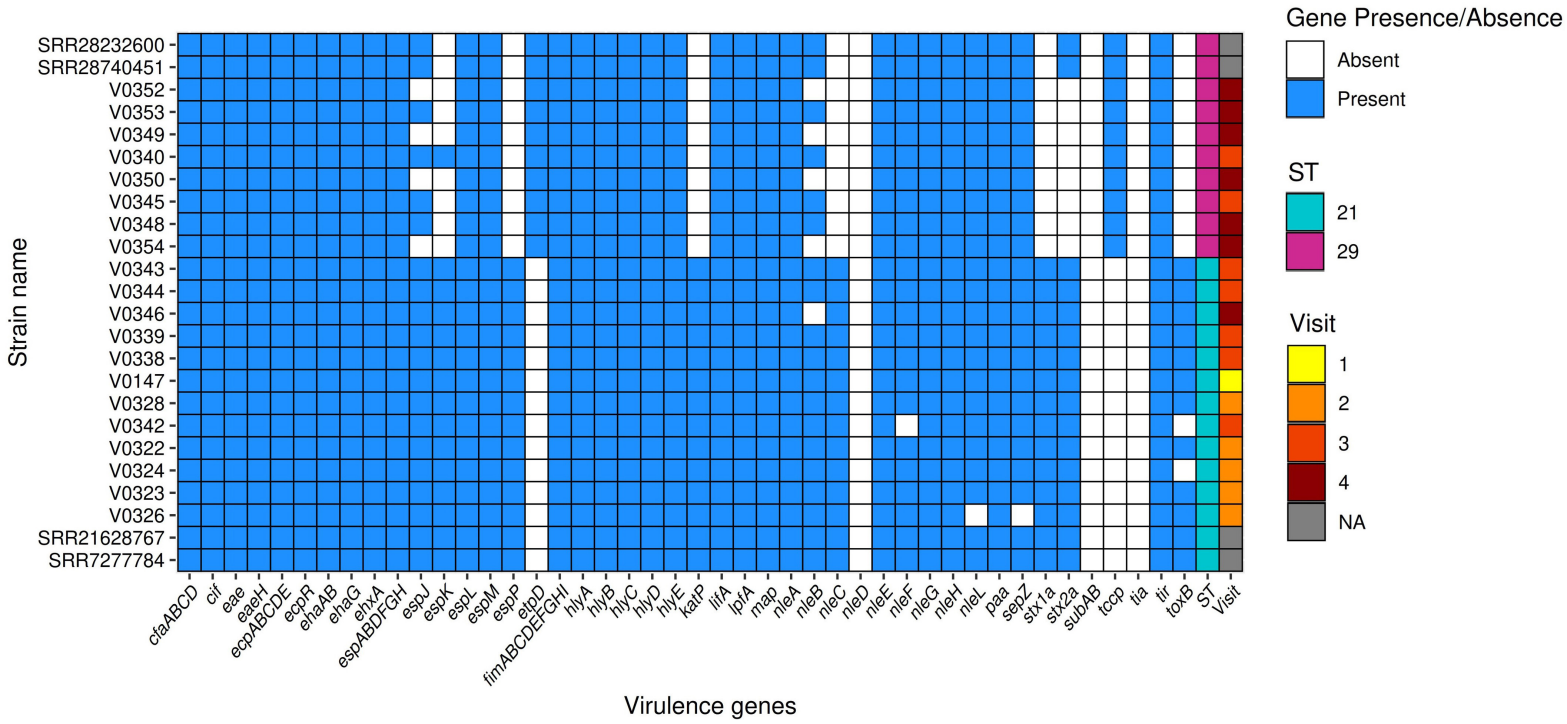
Presence/absence of selected virulence genes in *Escherichia coli* O26 study isolates. Study isolates are denoted with “V”. Isolates SRR7277784, SRR21628767, SRR28740451, are SRR28232600 are publicly available STEC O26 derived from humans, sequence type 21 and 29, respectively, included for comparison purposes. Sequence type (ST) is shown in the second to last column to the right, visit number is shown in the last column to the right.

**Figure 4:**
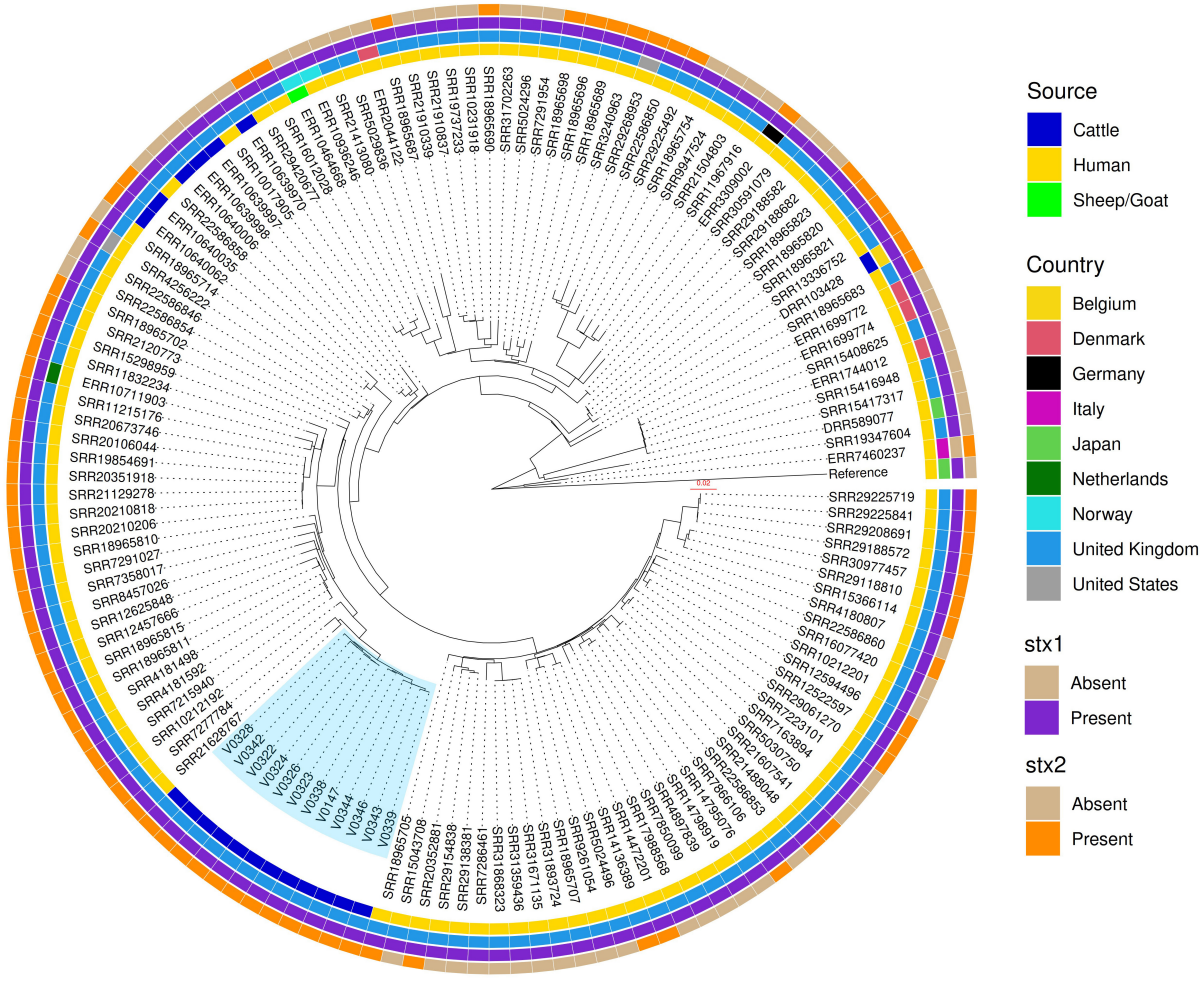
Sequence type 21 *Escherichia coli* O26 single nucleotide polymorphism maximum likelihood phylogenetic tree. RAxML-NG tree made from Snippy core.full.aln filtered with SNP-sites. Metadata of each isolate is displayed in colored rings (inwards to outwards): source, country, Shiga toxin 1 presence/absence, and Shiga toxin 2 presence/absence. *E. coli* O26 ST21 isolated in this study are highlighted in blue.

A core genome SNP-based maximum likelihood phylogenetic tree of the farm isolates showed two distinct populations of *E. coli* O26:H11 grouped by sequence type, separating ST21 from ST29 (Figure 2). The ST21 *E. coli* O26:H11 all carried *stx1a, stx2a* and *eae* genes (Figure 3), and belonged to clonal complex CC29. The SNP distance matrix of these STEC O26:H11 ST21 isolates showed the number of SNPs ranged from 3 to 29 across the four visits (Supplementary Table S4); of these pairwise SNP distances, 21 out of 66 pairs (31.8%) were clonal ( ≤ 10 SNPs), 33 pairs (50.0%) were very closely related (>10 and ≤ 20 SNPs), and 12 (18.2%) pairs were closely related (>20 and ≤ 30 SNPs; Supplementary Table S6). Most clonal isolates were spread over visits 1, 2, and 3, the average pairwise SNP distances between Visit 1 and subsequent visits were: Visit 1 → 2 (10 SNPs), Visit 1 → 3 (9 SNPs) and Visit 1 → 4 (12 SNPs). Isolate V0346 from visit 4 showed clonal relationships with three isolates from visit 3 (V0339, V0343, and V0344; Figure 2, Supplementary Table S4). Since a clonal STEC O26 ST21 population was apparent from comparison of isolates from Visit 1 → 2 → 3, Visit 1 was considered an appropriate starting point for the year-long pilot study on persistence. To further understand how these farm isolates relate to wider global ST21 population, we extended the phylogenetic analysis to include publicly available ST21 *E. coli* O26 genomes (study isolates, *n* = 12; global isolates *n* = 126; Figure 4). The farm isolates formed a distinct sub-cluster adjacent to a branch of STEC O26s associated with human infection from the UK; but they were at least 51 (SRR7277784) and 61 SNPs (SRR21628767) from the closest human isolates (Supplementary Tables S8, S9; isolate metadata provided in Supplementary Table S3). The estimated genome sizes of our ST21s ranged from 5.3 to 5.5 Mbp, being slightly smaller than human STEC O26s with 5.6 Mbp genomes.

The other *E. coli* O26:H11 population, ST29, were all negative for Shiga toxin genes but carried the intimin gene. The number of SNPs among these non-STEC O26:H11 ranged from 0–13 (Supplementary Table S4), and the pairwise SNP distance matrix showed 23/28 (82.1%) pairs were clonal i.e., ≤ 10 SNPs, and 5 (17.8%) pairs were very closely related. Phylogenetic comparison of the farm ST29 *E. coli* O26:H11 with publicly available isolates (study isolates, *n* = 8; global isolates, *n* = 108; Supplementary Figure S1) showed our UK-farm isolates grouped on a sub-cluster close to three human strains of ST29 STEC O26:H11 from the UK but were at least 29 SNPs distant from these isolates.

During the first and second visits, all recovered isolates were STEC O26:H11 ST21. In the third visit, five isolates were STEC O26:H11 ST21, while two were non-STEC O26:H11 ST29. During the final visit, only one STEC O26:H11 ST21 was recovered, alongside seven non-STEC O26:H11 ST29 (Table 2). Additionally, two non-O26 STECs including O145:H12 ST101 and O130:H53 ST297 (Table 2) were also recovered during visit 4.

Among the age groups sampled during the longitudinal visits (visit 2, 3, and 4), only STEC O26:H11 belonging to ST21 were isolated from adults. However, among the youngstock samples, both STEC and non-STEC O26:H11 were isolated.

#### 3.4.2 Virulence genes characterization

**Figure 5:**
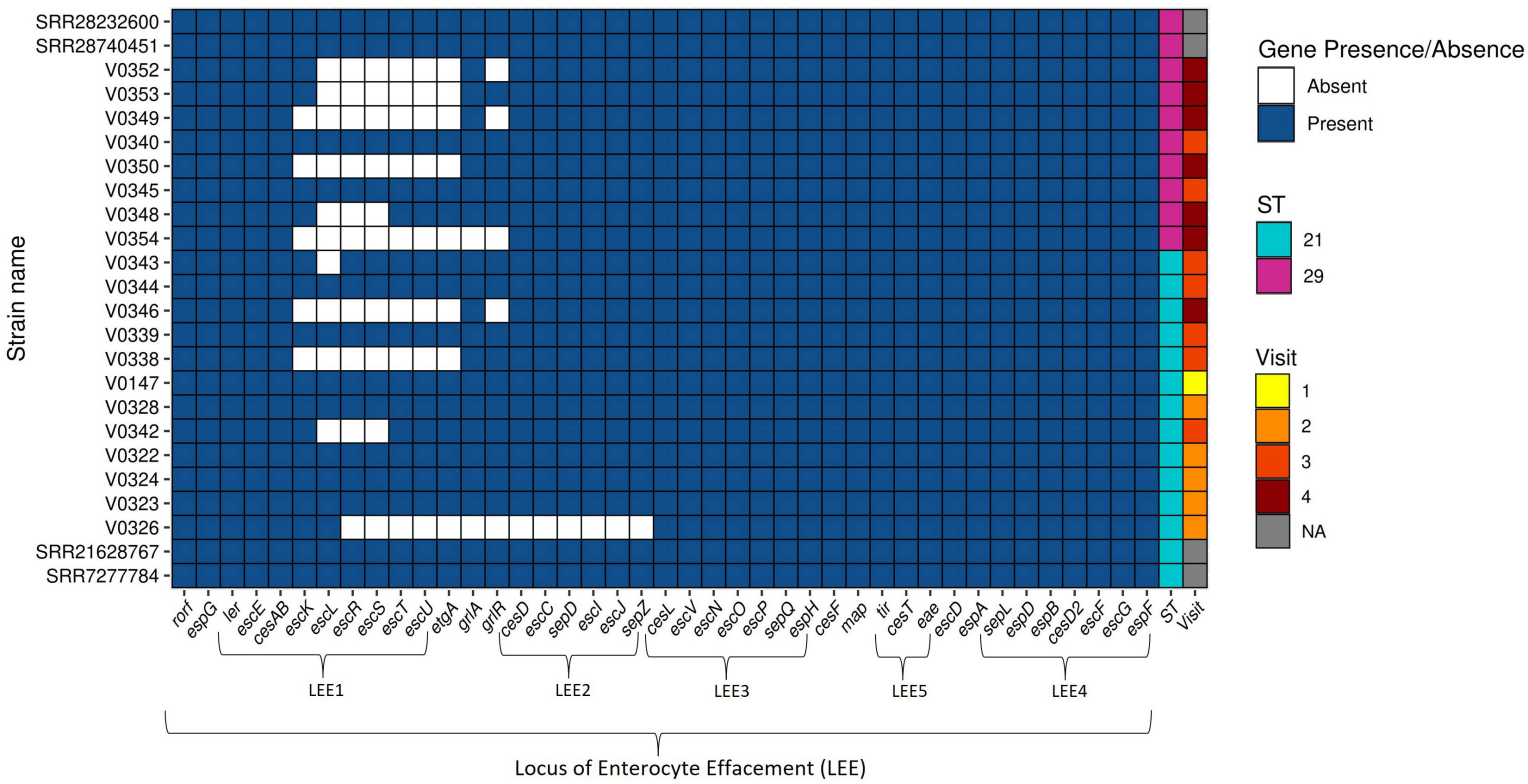
Presence of Locus of enterocyte effacement (LEE) genes in *Escherichia coli* O26 study isolates. Study isolates are denoted with “V”. Isolates SRR7277784, SRR21628767, SRR28740451, are SRR28232600 are publicly available STEC O26 derived from humans, sequence type 21 and 29, respectively, included for comparison purposes. LEE operon 1, 2, 3, 4, and 5 genes are indicated. Sequence type (ST) is shown in the second to last column to the right, visit number is shown in the last column to the right.

Characterization of virulence genes associated with STEC pathotypes revealed LEE genes in all *E. coli* O26. A complete LEE was present in seven ST21 STEC O26:H11 isolates: V0147, V0322, V0323, V0324, V0328, V0339, and V0344 (one from initial visit, four from visit 2, and two from visit 3); and two ST29 non-STEC isolates from visit 3: V0345 and V0340 (Figure 5). The remaining isolates lacked between 1 and 13 LEE genes, predominantly within LEE 1 and LEE 2 operons. Distantly related public O26:H11 ST21 (SRR21628767 and SRR7277784) and O26:H11 ST29 (SRR28232600 and SRR28740451) isolates included for comparison, showed that the farm ST21 isolates harboring the complete LEE had the same selected virulence gene profile as that of SRR21628767 and SRR7277784 (Figure 3), both of which are STEC O26:H11 ST21 isolates from human clinical cases in the UK.

Virulence genes related to adhesion were detected, with *eae* and *eaeH*, encoding the intimin-like adhesin, present in all recovered O26:H11 isolates (Figure 3). Other notable virulence-associated adhesion/colonization genes identified in some or all the study isolates include: *ehaAB, ehaG, lpfA, paa, tir*, and *toxB* (Figure 3). Beside *stx*, toxin genes *hlyA* and *hlyE*, encoding α-haemolysin and haemolysin E, respectively, and *ehxA* encoding enterohaemolysin were found in all O26:H11 isolates. Plasmid indicator genes, *katP* and *espP* (Etcheverria and Padola, 2013), were identified in all ST21 STEC O26:H11 but not in the ST29 O26:H11 isolates. Our *E. coli* O26:H11 isolates also grouped within the global sequence type lineages, distinguished by the following plasmid gene profiles, *ehxA*+*/katP*+*/espP*+*/etpD*- for ST21 and *ehxA*+*/katP-/espP-/etpD*+ for ST29 (Ogura et al., 2017).

Non-LEE encoded (Nle) effector proteins were also observed in the STEC O26:H11 study isolates (Figure 5); immune modulating effector genes *nleB, nleC, nleE*, and *nleH* were detected in the majority of ST21 isolates, but *nleD* was absent.

## 4 Discussion

As non-O157 STEC human infections including O26, continue to increase (Vishram et al., 2021), understanding their presence at primary production is crucial to inform on potential zoonotic risk. In this year-long pilot study, STEC O26:H11 occurrence was low (5.6%), yet the serogroup was consistently detected across all visits, suggesting persistence on this farm. For context, a 2001 Scottish farm study reported higher positivity in calf feces (16.5%, *n* = 110/664), and 5.8% in dam feces (*n* = 5/86; Pearce et al., 2004), while abattoir-based northern Italian data showed increased detection of STEC O26 O26:H11 from 0.5% (2007) to 3.8% (2015; Bonardi et al., 2007, 2015). We detected STEC O26:H11 on only one of 11 dairy farms in a preliminary screening (data not shown). A 2014–2015 study of Scottish cattle herds reported 15 of 110 herds positive for STEC O26 (Hoyle et al., 2021), consistent with the predominance of O26 serogroup among non-O157 STEC human infections in Scotland (Pearce et al., 2004; Callaway et al., 2009). A notable difference across studies is the detection methodology, which can influence occurrence (Martinez-Castillo and Muniesa, 2014). Moreover, STECs represent only a minority (< 0.01%−1.0%) of the *E. coli* population present in the cattle gut and feces (Callaway et al., 2009; Wells et al., 2014). However, Hoyle et al. (2021) also suggested the increased detection of *E. coli* O26 they observed may reflect a true increase in Scottish herd carriage over time.

We observed more STEC O26:H11 detection during visit 3 compared to visit 4, which was significant by Chi-square test. Bayesian analysis suggested lower odds of detection during visit 4, although with substantial uncertainty due to the low number of positive events. These patterns may be suggestive of visit-specific or sampling effects rather than true seasonal effects, as the visits were not evenly spaced to reflect seasonal transitions. Although seasonal variations in STEC shedding have been previously documented, the evidence across studies are inconsistent (Dewsbury et al., 2015; Hoyle et al., 2021). Overall, while the reduction in detection between visits is apparent, further conclusive evidence is required, highlighting the value of Bayesian analysis in capturing uncertainty and guiding further investigation in low-event datasets.

Despite the study farm being a closed herd, continual detection of STEC O26:H11 may reflect persistence in the cattle, its environment or regular re-introduction through carriers such as wild animals, humans, or farm vehicles. The occurrence of STEC involving multiple serogroups from food-producing animals and their products has been reported previously (Zhang et al., 2022; Li et al., 2016). We observed higher proportion of STEC O26:H11 ST21 in youngstock than in adults, and non-O26 STEC of serogroups O130 and O145, carrying *stx1a* were also detected in youngstock, suggestive of higher susceptibility of younger animals to STEC carriage but not sufficient to infer causal age effect (Baines and Erb, 2013; Nielsen et al., 2002; Zhao et al., 2013). Although, limited to four sampling points, the emergence of STEC serotype O130:H53 and O145:H12 during the last visit suggests possible turnover of isolates or ongoing microbial influx, an indication that the farm is an “open microbial system.” Despite our isolation method being tailored to serogroup O26 using IMS, recovery of other STEC serogroups highlights both the open microbial system of the farm and non-specificity of IMS, as we have noted previously with other STEC O-antigens (Kirchner et al., 2019).

We noted, environmental samples positive for STEC O26:H11 were found in wet areas, aligning with reports of *E. coli* survival in moist environments (Zweifel et al., 2013; Bonanno et al., 2015). Although, our study did not evaluate disinfectant efficacy, thorough cleaning, disinfection, and drying of animal housing prior to introducing the new calves is prudent given that STEC infection can occur soon after birth (Fernández et al., 2012). In the UK, the use of Government-approved disinfectants at appropriate concentrations is standard practice; suboptimal concentration has been associated with disinfectant tolerance in O157:H7 (Kirchner et al., 2024), suggesting plausible, but untested parallels for O26:H11.

A greater proportion of pooled samples tested positive for STEC O26:H11 than individual samples, possibly because each pooled sample represented a higher number of animals. However, interpretation is limited both statistically and methodologically, as over twice as many pooled samples were collected and pooled samples represent two different sampling approaches (boot swab and pooled pats), which may vary in sensitivity. Sanderson et al. (2005) found pooling of feces to be detrimental to STEC detection at low prevalence compared to individual samples, but similar at high prevalence.

Overall, the effect of housing on STEC O26:H11 detection was inconclusive due to low positivity, *ad hoc* sampling and limited sample size, reducing statistical power to identify indoor-outdoor differences. Nevertheless, our modeling showed statistically significant reduced odds of STEC O26 detection at outdoor sites, although it requires cautious interpretation. This may reflect lower pathogen persistence in open environments, dilution effects, or higher infection of the youngstock which were typically indoors (Van Elsas et al., 2011; Browne et al., 2018; Henry et al., 2019). An observation of note was detection of STEC O26 in a single barn at all visits except one, this may have been by chance or conditions during visit 2 were less favorable for STEC O26 shedding.

Non-STEC O26:H11 ST29 was first isolated during visit 3 in youngstock at pasture and detected again during the final visit, suggesting a plausible persistence among youngstock housed with positive animals approximately 0.8 km apart. One possible explanation is initial acquisition at pasture, followed by circulation after animals were moved indoors for winter housing. Shared grazing areas on pasture may facilitate initial exposure through ingestion of contaminating feces. The increased detection during the third and final visits, is also compatible with conditions that facilitate onward transmission including increased animal density, close contact, and shared housing facilities (Henry et al., 2019; Venegas-Vargas et al., 2016; Frank et al., 2008). A similar pattern was not observed for STEC O26 ST21, possibly linked to the introduction of and competition with the O26:H11 ST29 population.

WGS permits in-depth genetic characterization of pathogens, and when applied to STEC, enables detection of *stx* gene variants, serotyping, LEE, prophages, and virulence-associated genes. Phylogenetic examination of the farm isolates showed ST21 O26:H11 persisted on the farm from the first to the final visit. Initially the population was mostly clonal with some divergence, as the study progressed many SNP-distances among the ST21 isolates began to increase. Plausible explanations for this observation could be within-farm isolates variation, or repeated introduction or phage-mediated recombination (Dallman et al., 2021) as well as uncertainty of its presence prior to our study. Additionally, as already mentioned, ST29 O26:H11 emerged later in the study and spread clonally, possibly following its introduction on farm and subsequently competing with ST21 O26:H11. An important aspect of STEC phylogeny during epidemiological investigations is genetic distance between human and animal isolates. Cattle are recognized sources of STEC O26 (Jenkins et al., 2003; Pearce et al., 2004; Hoyle et al., 2021), and in our study, the phylogenetic analysis indicates that a population of O26:H11 cattle isolates from the farm were clonal and distinct from globally reported cattle isolates. However, they were relatively closer, but not genetically linked, to human STEC O26:H11 isolates from the UK. Ogura et al. (2017) provided a phylogenetic overview of *E. coli* O26:H11, where cattle and human isolates were indistinguishable indicating the zoonotic potential of cattle isolates. Our STEC O26:H11 isolates fell within a predominant UK cluster of human ST21 strains, while *stx*-negative ST29 isolates clustered within a European cluster harboring *stx2* from humans in the UK, France, Denmark, Germany, Belgium, Netherlands, and Norway, and cattle from Germany.

Our isolates match the expected plasmid gene profile (*ehxA/katP/espP/etpD*) according to their ST (ST21 or ST29), aligning with global lineages (Ogura et al., 2017; Bielaszewska et al., 2013; Hoyle et al., 2023). Notably, all our ST21 and ST29 isolates belong to the STEC CC29 (Rodwell et al., 2023), although the ST29 lacked *stx* genes. Over the last decade, there has been a rise in human STEC cases causing HUS in Great Britain, involving CC29, with most STEC O26:H11 harboring *stx2a* alone or in combination with *stx1a* (Rodwell et al., 2023). According to FAO and WHO (2018), the genotype *eae, stx1a* and stx2a, as seen in our ST21 isolates, falls within the highest risk category for HUS. In Germany, O26:H11 harboring *stx2a* was most associated with HUS, accounting for 33% cases (Fruth et al., 2024). The LEE pathogenicity island (35.6 kb) is crucial for STEC in causing gut attaching and effacing lesions (Franzin and Sircili, 2015). In our study, nine O26 isolates harbored the complete LEE, with variable composition in other isolates. Hoyle et al. (2023) also found absence of some LEE genes in STEC O26 from humans and cattle in the UK. Our ST29 isolates showed similar virulence gene profile (i.e., excluding LEE) to the public isolates SRR28232600 and SRR28740451, associated with human infection in the UK, both of which harbored *stx2a*. Since our ST29 isolates lacked *stx* genes and are unlikely to cause severe disease, they may still pose a risk to humans through other virulence factors including non-LEE-encoded gene, as reported in *stx*-negative EHEC O26:H11 (Bonanno et al., 2015; Zweifel et al., 2013). Also, *nleD*, involved in immune evasion by suppressing the JNK signaling pathway and IL-8 secretions (Baruch et al., 2011), was not detected in our isolates consistent with previous studies, including from the UK, where the majority of isolates did not harbor *nleD* but other *nle* genes were identified (Hoyle et al., 2023).

Overall the detection of STEC O26 in the farm was not high, however, its presence and persistence in cattle and/or their environment poses a continuous public health risk (Ward et al., 2025), particularly considering their low infective dose (10-100 organisms) which is similar to STEC O157:H7 (Dallman et al., 2021). Genomic characterization of STEC from animal reservoirs is important for understanding their within-farm clonality and persistence of circulating strains, which remains critical to support epidemiological investigations involving human outbreaks of STEC infection.

This pilot study was constrained by limited statistical power, as several predictors were not independent such as settings, locations, sample type, visit, and age, as well as limited timepoints to assess seasonal effects, restricting ability to identify strong effects and significant risk factors. Nevertheless, our study provides valuable initial insights which can be used for further investigations in future. Further research would benefit from longitudinal sampling of traceable animal cohorts across production cycles, using balanced sampling plans across sample types and time points, and cost effective approaches that may enhance evidence-based surveillance (Sora et al., 2024).

## Data Availability

The datasets presented in this study can be found in NCBI BioProject (https://www.ncbi.nlm.nih.gov/bioproject), accession number: PRJNA1178587.
