# Supplementary material for: Genomic insights into within-farm persistence and global phylogenetic relatedness of Shiga toxin-producing Escherichia coli O26 in a dairy cattle farm in the UK
Source: Front Microbiol. 2026 Apr 9;17:1786881. doi: 10.3389/fmicb.2026.1786881 (PMC13102674; doi:10.3389/fmicb.2026.1786881)
Supplement: Supplementary file 1 [file Data_Sheet_1.pdf]

Supplementary

Initial screening visit (visit 1)

During the initial farm visit in November 2018, screening was conducted to detect the presence of STEC O26, O103, O111 and O145, known to be associated with human disease cases in the EU and previously isolated from farm animals. Only youngstock samples were collected during the screening visit because this age group is associated with higher risk of STEC carriage and shedding. Sampling comprised collecting 9 faecal samples including pooled faeces (n=6) and boot swabs (n=3). One swab was taken from three separate groups of 4-6 months old weaned-calves in adjacent pens in an open-sided building on the main farm site (S1). The pooled faecal samples were collected from a single group of 9–15-month-old youngstock at pasture (S2), approximately 0.5 km from the main farm site. Additionally, an environmental swab was collected from the vicinity of a water trough in this pasture. Details in Supplementary Table 1.

Samples were enriched in 90 mL BPW at 41.5°C for 24 hours. IMS was performed on 1 mL of broth enrichments using pools of Dynabeads for serogroups O26, O103, O111 and O145 (20 µL of each Dynabead). The IMS eluate was grown on sorbitol McConkey agar supplemented with cefixime and potassium tellurite and Chromagar ECC. A loopful of growth were picked using a 1 µL loop from each plate, suspended in 200 µL of sterile molecular-grade water, and boiled at 100°C for 15 minutes for PCR screening of the target serogroups and Shiga toxin genes.

Supplementary Figure S1. Sequence type 29 *Escherichia coli* O26 single nucleotide polymorphism maximum likelihood phylogenetic tree.

RAxML-NG tree made from Snippy core.full.aln filtered with SNP-sites. Metadata of each isolate is displayed in coloured rings (inwards to outwards): source, country, Shiga toxin 1 presence/absence, and Shiga toxin 2 presence/absence. *E. coli* O26 ST29 isolated in this study are highlighted in blue.

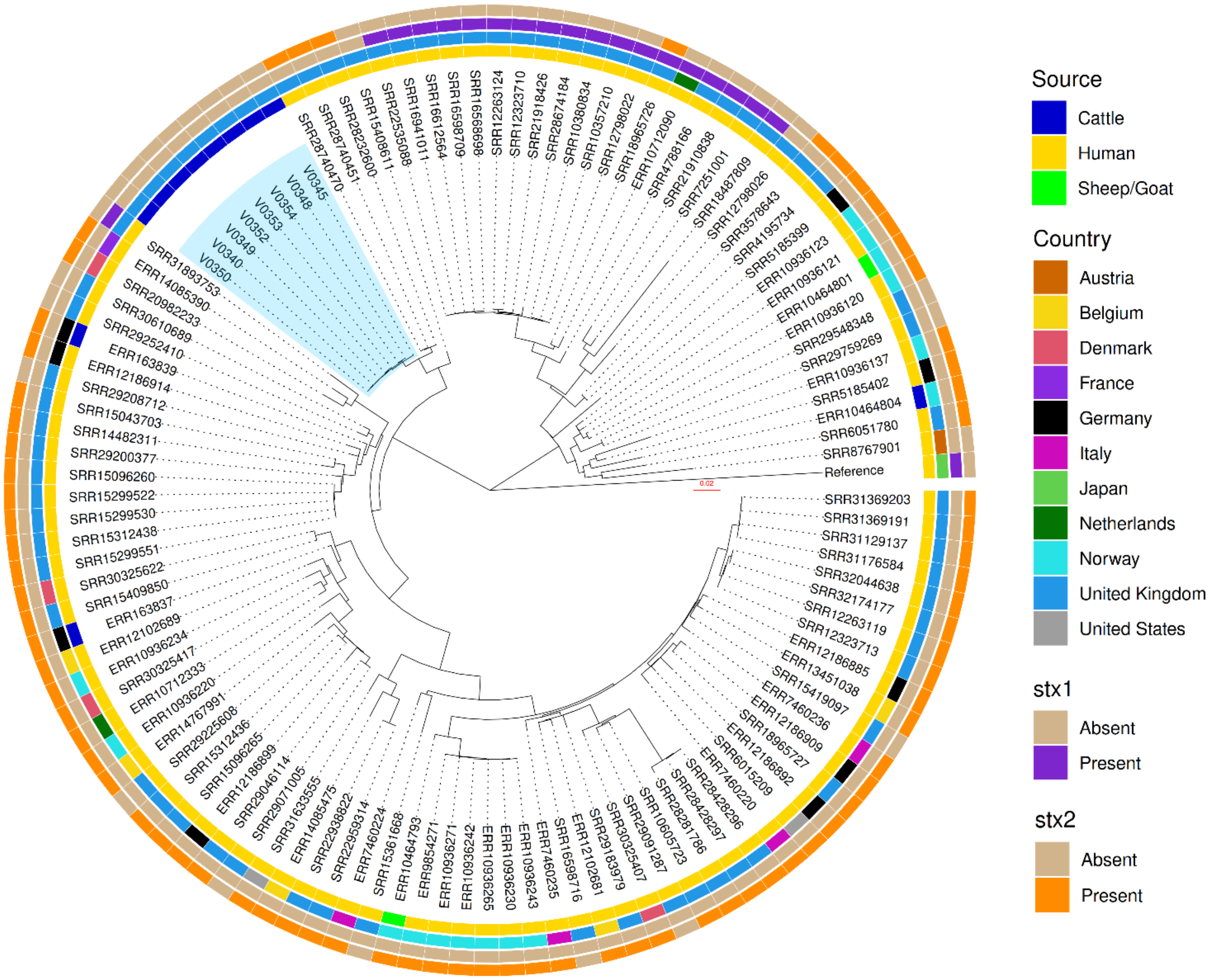

Supplementary Table S1. Date and location of herd at each sampling visit

	Visit 1 (Screening visit)	Visit 2	Visit 3	Visit 4
**Date**	Nov-2018	Jun-2019	Oct-2019	Dec-2019
**Location of adults**	Housed	Pasture	Pasture	Housed
**Location of youngstock (>6 months old)**	Pasture	Pasture	Pasture	Housed
**Location of youngstock (<6 months old)**	Housed	Housed	Housed	Housed

Supplementary Table S2. Sampling site identification, description, and sampling pattern during the study

Sampling site ID	Site description	Visit 1 (screening)	Visit 2	Visit 3	Visit 4
S1	Indoor housing (main farm site)	✓	✓	✓	✓
S2	Pasture	✓	✓		
S3	Pasture		✓		
S4	Pasture		✓		
S5	Pasture		✓	✓	

S6	Pasture		✓	✓	
S7	Pasture		✓		
S8	Pasture			✓	
S9	Pasture			✓	
S10	Pasture			✓	
S11	Pasture			✓	
S12	Pasture			✓	
S13	Pasture			✓	
S14	Indoor housing				✓

Supplementary Table S3. *Escherichia coli* O26 isolates metadata

**Label**	**Source Type**	**Country**	**Year**	**ST**	***stx1***	***stx2***	**Source**	**Accession or SRA**
SRR2120773	Human	United Kingdom	2010	21	Present	Present	EnteroBase	SRR2120773
SRR9261054	Human	United Kingdom	2019	21	Present	Absent	EnteroBase	SRR9261054
SRR15408625	Human	United Kingdom	2017	21	Present	Absent	EnteroBase	SRR15408625
SRR15416948	Human	United Kingdom	2017	21	Present	Absent	EnteroBase	SRR15416948
SRR15417317	Human	United Kingdom	2017	21	Present	Absent	EnteroBase	SRR15417317
SRR17988568	Human	United Kingdom	2022	21	Present	Absent	EnteroBase	SRR17988568
SRR9947524	Human	United Kingdom	2019	21	Present	Absent	EnteroBase	SRR9947524
ERR3309002	Human	Germany	2016	21	Present	Present	EnteroBase	ERR3309002
SRR18965810	Human	United Kingdom	2008	21	Present	Present	EnteroBase	SRR18965810

SRR18965811	Human	United Kingdom	2015	21	Present	Present	EnteroBase	SRR18965811
SRR18965815	Human	United Kingdom	2017	21	Present	Present	EnteroBase	SRR18965815
SRR18965820	Human	United Kingdom	2010	21	Present	Present	EnteroBase	SRR18965820
SRR18965821	Human	United Kingdom	2010	21	Present	Present	EnteroBase	SRR18965821
SRR18965823	Human	United Kingdom	2010	21	Present	Present	EnteroBase	SRR18965823
SRR18965754	Human	United Kingdom	2012	21	Present	Absent	EnteroBase	SRR18965754
SRR18965683	Human	United Kingdom	2009	21	Present	Present	EnteroBase	SRR18965683
SRR18965687	Human	United Kingdom	2005	21	Present	Present	EnteroBase	SRR18965687
SRR18965689	Human	United Kingdom	2006	21	Present	Present	EnteroBase	SRR18965689
SRR18965690	Human	United Kingdom	2011	21	Present	Present	EnteroBase	SRR18965690
SRR18965696	Human	United Kingdom	2014	21	Present	Present	EnteroBase	SRR18965696
SRR18965698	Human	United Kingdom	2010	21	Present	Present	EnteroBase	SRR18965698
SRR18965702	Human	United Kingdom	2013	21	Present	Present	EnteroBase	SRR18965702
SRR18965705	Human	United Kingdom	2010	21	Present	Present	EnteroBase	SRR18965705
SRR18965707	Human	United Kingdom	2017	21	Present	Absent	EnteroBase	SRR18965707
SRR18965714	Human	United Kingdom	2006	21	Present	Absent	EnteroBase	SRR18965714
SRR19347604	Human	United Kingdom	2022	21	Present	Absent	EnteroBase	SRR19347604
ERR7460237	Human	Italy	2015	21	Absent	Present	EnteroBase	ERR7460237
SRR19737233	Human	United Kingdom	2022	21	Present	Absent	EnteroBase	SRR19737233
SRR10017905	Human	United Kingdom	2019	21	Present	Absent	EnteroBase	SRR10017905
SRR19854691	Human	United Kingdom	2022	21	Present	Present	EnteroBase	SRR19854691
SRR20210818	Human	United Kingdom	2022	21	Present	Present	EnteroBase	SRR20210818
SRR20210206	Human	United Kingdom	2022	21	Present	Present	EnteroBase	SRR20210206
SRR20106044	Human	United Kingdom	2022	21	Present	Present	EnteroBase	SRR20106044
SRR20352881	Human	United Kingdom	2022	21	Present	Present	EnteroBase	SRR20352881
SRR20351918	Human	United Kingdom	2022	21	Present	Present	EnteroBase	SRR20351918
SRR20673746	Human	United Kingdom	2022	21	Present	Present	EnteroBase	SRR20673746
ERR2044122	Human	Denmark	2016	21	Present	Absent	EnteroBase	ERR2044122
SRR10212192	Human	United Kingdom	2019	21	Present	Present	EnteroBase	SRR10212192
SRR10212201	Human	United Kingdom	2019	21	Present	Absent	EnteroBase	SRR10212201
SRR10231918	Human	United Kingdom	2019	21	Present	Absent	EnteroBase	SRR10231918

SRR21129278	Human	United Kingdom	2022	21	Present	Present	EnteroBase	SRR21129278
SRR21488048	Human	United Kingdom	2022	21	Present	Present	EnteroBase	SRR21488048
SRR21413080	Human	United Kingdom	2022	21	Present	Absent	EnteroBase	SRR21413080
SRR21504803	Human	United Kingdom	2022	21	Present	Absent	EnteroBase	SRR21504803
SRR21607541	Human	United Kingdom	2022	21	Present	Present	EnteroBase	SRR21607541
SRR21628767	Human	United Kingdom	2022	21	Present	Present	EnteroBase	SRR21628767
SRR3240963	Human	United Kingdom	2014	21	Present	Present	EnteroBase	SRR3240963
SRR21910837	Human	United Kingdom	2022	21	Present	Absent	EnteroBase	SRR21910837
SRR21910339	Human	United Kingdom	2022	21	Present	Absent	EnteroBase	SRR21910339
SRR22586846	Human	United Kingdom	2020	21	Present	Absent	EnteroBase	SRR22586846
SRR22586850	Human	United Kingdom	2019	21	Present	Present	EnteroBase	SRR22586850
SRR22586853	Human	United Kingdom	2019	21	Present	Absent	EnteroBase	SRR22586853
SRR22586854	Human	United Kingdom	2020	21	Present	Absent	EnteroBase	SRR22586854
SRR22586858	Human	United Kingdom	2020	21	Present	Absent	EnteroBase	SRR22586858
SRR22586860	Human	United Kingdom	2020	21	Present	Present	EnteroBase	SRR22586860
SRR4180807	Human	United Kingdom	2015	21	Present	Absent	EnteroBase	SRR4180807
SRR4181498	Human	United Kingdom	2015	21	Present	Present	EnteroBase	SRR4181498
SRR4181592	Human	United Kingdom	2015	21	Present	Present	EnteroBase	SRR4181592
SRR4256222	Human	United States	2016	21	Present	Present	EnteroBase	SRR4256222
ERR10640062	Cattle	United Kingdom	2014	21	Present	Present	EnteroBase	ERR10640062
ERR10640035	Cattle	United Kingdom	2014	21	Present	Present	EnteroBase	ERR10640035
ERR10640006	Cattle	United Kingdom	2003	21	Present	Absent	EnteroBase	ERR10640006
ERR10639998	Cattle	United Kingdom	2003	21	Present	Absent	EnteroBase	ERR10639998
ERR10639997	Cattle	United Kingdom	2002	21	Present	Absent	EnteroBase	ERR10639997
ERR10639970	Cattle	United Kingdom	2002	21	Present	Absent	EnteroBase	ERR10639970
ERR10711903	Human	Netherlands	2017	21	Present	Present	EnteroBase	ERR10711903
ERR10936246	Human	Norway	2021	21	Present	Absent	EnteroBase	ERR10936246
SRR4897839	Human	United Kingdom	2016	21	Present	Absent	EnteroBase	SRR4897839
SRR5029836	Human	United Kingdom	2015	21	Present	Absent	EnteroBase	SRR5029836
SRR11215176	Human	United Kingdom	2020	21	Present	Present	EnteroBase	SRR11215176
ERR10464668	Sheep/Goat	Norway	2016	21	Present	Absent	EnteroBase	ERR10464668
SRR5030750	Human	United Kingdom	2015	21	Present	Absent	EnteroBase	SRR5030750

SRR5024296	Human	United Kingdom	2015	21	Present	Absent	EnteroBase	SRR5024296
SRR5024496	Human	United Kingdom	2016	21	Present	Absent	EnteroBase	SRR5024496
SRR11832234	Human	United Kingdom	2020	21	Present	Present	EnteroBase	SRR11832234
SRR11967916	Human	United Kingdom	2020	21	Present	Absent	EnteroBase	SRR11967916
SRR12457666	Human	United Kingdom	2020	21	Present	Present	EnteroBase	SRR12457666
SRR12522597	Human	United Kingdom	2020	21	Present	Present	EnteroBase	SRR12522597
SRR12594496	Human	United Kingdom	2020	21	Present	Present	EnteroBase	SRR12594496
SRR12625848	Human	United Kingdom	2020	21	Present	Present	EnteroBase	SRR12625848
SRR29061270	Human	United Kingdom	2013	21	Present	Present	EnteroBase	SRR29061270
SRR29118810	Human	United Kingdom	2023	21	Present	Present	EnteroBase	SRR29118810
SRR29138381	Human	United Kingdom	2023	21	Present	Absent	EnteroBase	SRR29138381
SRR29154838	Human	United Kingdom	2023	21	Present	Absent	EnteroBase	SRR29154838
SRR29188682	Human	United Kingdom	2023	21	Present	Absent	EnteroBase	SRR29188682
SRR29188582	Human	United Kingdom	2023	21	Present	Absent	EnteroBase	SRR29188582
SRR29188572	Human	United Kingdom	2023	21	Present	Present	EnteroBase	SRR29188572
SRR29208691	Human	United Kingdom	2023	21	Present	Present	EnteroBase	SRR29208691
SRR29225841	Human	United Kingdom	2023	21	Present	Present	EnteroBase	SRR29225841
SRR29225719	Human	United Kingdom	2023	21	Present	Present	EnteroBase	SRR29225719
SRR29225492	Human	United Kingdom	2023	21	Present	Present	EnteroBase	SRR29225492
SRR29268953	Human	United States	2024	21	Present	Present	EnteroBase	SRR29268953
SRR29420677	Human	United Kingdom	2024	21	Present	Present	EnteroBase	SRR29420677
DRR103428	Cattle	Belgium	2011	21	Present	Present	EnteroBase	DRR103428
DRR589077	Human	Japan	2021	21	Present	Absent	EnteroBase	DRR589077
SRR7163894	Human	United Kingdom	2017	21	Present	Absent	EnteroBase	SRR7163894
SRR7215940	Human	United Kingdom	2017	21	Present	Present	EnteroBase	SRR7215940
SRR7223101	Human	United Kingdom	2017	21	Present	Absent	EnteroBase	SRR7223101
SRR7277784	Human	United Kingdom	2015	21	Present	Present	EnteroBase	SRR7277784
SRR7286461	Human	United Kingdom	2017	21	Present	Absent	EnteroBase	SRR7286461
SRR7291027	Human	United Kingdom	2016	21	Present	Present	EnteroBase	SRR7291027
SRR7291954	Human	United Kingdom	2014	21	Present	Absent	EnteroBase	SRR7291954
SRR7358017	Human	United Kingdom	2015	21	Present	Present	EnteroBase	SRR7358017

SRR30591079	Human	United Kingdom	2024	21	Present	Absent	EnteroBase	SRR30591079
SRR30977457	Human	United Kingdom	2024	21	Present	Present	EnteroBase	SRR30977457
SRR7850099	Human	United Kingdom	2018	21	Present	Absent	EnteroBase	SRR7850099
SRR7866106	Human	United Kingdom	2018	21	Present	Present	EnteroBase	SRR7866106
SRR13336752	Human	United Kingdom	2014	21	Present	Present	EnteroBase	SRR13336752
SRR31359436	Human	United Kingdom	2024	21	Present	Absent	EnteroBase	SRR31359436
SRR31702263	Human	United Kingdom	2024	21	Present	Absent	EnteroBase	SRR31702263
SRR31671135	Human	United Kingdom	2024	21	Present	Absent	EnteroBase	SRR31671135
ERR1699772	Human	Denmark	2007	21	Present	Absent	EnteroBase	ERR1699772
ERR1699774	Human	Denmark	2007	21	Present	Absent	EnteroBase	ERR1699774
ERR1744012	Human	Denmark	2007	21	Present	Absent	EnteroBase	ERR1744012
SRR8457026	Human	United Kingdom	2019	21	Present	Present	EnteroBase	SRR8457026
SRR31868323	Human	United Kingdom	2024	21	Present	Absent	EnteroBase	SRR31868323
SRR31893724	Human	United Kingdom	2024	21	Present	Absent	EnteroBase	SRR31893724
SRR14472201	Human	United Kingdom	2021	21	Present	Present	EnteroBase	SRR14472201
SRR14136389	Human	United Kingdom	2021	21	Present	Present	EnteroBase	SRR14136389
SRR14795076	Human	United Kingdom	2021	21	Present	Absent	EnteroBase	SRR14795076
SRR14798919	Human	United Kingdom	2021	21	Present	Absent	EnteroBase	SRR14798919
SRR15043708	Human	United Kingdom	2021	21	Present	Absent	EnteroBase	SRR15043708
SRR15366114	Human	United Kingdom	2021	21	Present	Present	EnteroBase	SRR15366114
SRR15298959	Human	United Kingdom	2021	21	Present	Present	EnteroBase	SRR15298959
SRR16012028	Human	United Kingdom	2021	21	Present	Present	EnteroBase	SRR16012028
SRR16077420	Human	United Kingdom	2021	21	Present	Absent	EnteroBase	SRR16077420
SRR15408611	Human	United Kingdom	2018	29	Absent	Absent	EnteroBase	SRR15408611
SRR15409850	Human	United Kingdom	2017	29	Absent	Present	EnteroBase	SRR15409850
SRR15419097	Human	United Kingdom	2015	29	Absent	Absent	EnteroBase	SRR15419097
SRR16568698	Human	United Kingdom	2021	29	Present	Absent	EnteroBase	SRR16568698
SRR16598709	Human	United Kingdom	2021	29	Present	Absent	EnteroBase	SRR16598709
SRR16598716	Human	United Kingdom	2021	29	Absent	Absent	EnteroBase	SRR16598716
SRR16612564	Human	United Kingdom	2021	29	Present	Absent	EnteroBase	SRR16612564
SRR16941011	Human	United Kingdom	2021	29	Present	Absent	EnteroBase	SRR16941011
ERR163837	Cattle	Germany	2008	29	Absent	Present	EnteroBase	ERR163837

ERR163839	Cattle	Germany	2007	29	Absent	Present	EnteroBase	ERR163839
SRR18487809	Human	United Kingdom	2022	29	Present	Absent	EnteroBase	SRR18487809
SRR18965726	Human	United Kingdom	2014	29	Present	Present	EnteroBase	SRR18965726
SRR18965727	Human	United Kingdom	2015	29	Absent	Present	EnteroBase	SRR18965727
ERR7460236	Human	Italy	2015	29	Absent	Present	EnteroBase	ERR7460236
ERR7460235	Human	Italy	2015	29	Absent	Present	EnteroBase	ERR7460235
ERR7460224	Human	Italy	2013	29	Absent	Present	EnteroBase	ERR7460224
ERR7460220	Human	Italy	2008	29	Absent	Present	EnteroBase	ERR7460220
ERR9854271	Human	Norway	2019	29	Absent	Present	EnteroBase	ERR9854271
SRR20982233	Human	Denmark	2022	29	Absent	Present	EnteroBase	SRR20982233
SRR3578643	Human	United Kingdom	2015	29	Absent	Present	EnteroBase	SRR3578643
SRR10357210	Human	United Kingdom	2019	29	Present	Absent	EnteroBase	SRR10357210
SRR10380834	Human	United Kingdom	2019	29	Present	Absent	EnteroBase	SRR10380834
SRR21910838	Human	United Kingdom	2022	29	Present	Absent	EnteroBase	SRR21910838
SRR21918426	Human	United Kingdom	2022	29	Present	Absent	EnteroBase	SRR21918426
SRR10605723	Human	United Kingdom	2019	29	Absent	Present	EnteroBase	SRR10605723
SRR22535088	Human	United Kingdom	2021	29	Present	Absent	EnteroBase	SRR22535088
SRR22938822	Human	United Kingdom	2022	29	Absent	Present	EnteroBase	SRR22938822
SRR22959314	Human	United Kingdom	2022	29	Absent	Present	EnteroBase	SRR22959314
SRR4195734	Human	United Kingdom	2015	29	Absent	Present	EnteroBase	SRR4195734
ERR10712333	Human	Netherlands	2020	29	Absent	Present	EnteroBase	ERR10712333
ERR10712090	Human	Netherlands	2018	29	Present	Absent	EnteroBase	ERR10712090
ERR10936271	Human	Norway	2021	29	Absent	Present	EnteroBase	ERR10936271
ERR10936265	Human	Norway	2021	29	Absent	Present	EnteroBase	ERR10936265
ERR10936243	Human	Norway	2021	29	Absent	Present	EnteroBase	ERR10936243
ERR10936242	Human	Norway	2021	29	Absent	Present	EnteroBase	ERR10936242
ERR10936234	Human	Norway	2021	29	Absent	Present	EnteroBase	ERR10936234
ERR10936230	Human	Norway	2021	29	Absent	Present	EnteroBase	ERR10936230
ERR10936220	Human	Norway	2021	29	Absent	Present	EnteroBase	ERR10936220
ERR10936137	Human	Norway	2014	29	Absent	Present	EnteroBase	ERR10936137
ERR10936123	Human	Norway	2010	29	Absent	Present	EnteroBase	ERR10936123
ERR10936121	Human	Norway	2008	29	Absent	Present	EnteroBase	ERR10936121
ERR10936120	Human	Norway	2007	29	Absent	Present	EnteroBase	ERR10936120
SRR4788166	Human	United Kingdom	2016	29	Present	Absent	EnteroBase	SRR4788166
ERR12102681	Human	Belgium	2023	29	Absent	Present	EnteroBase	ERR12102681
ERR10464804	Cattle	Norway	2014	29	Absent	Present	EnteroBase	ERR10464804
ERR10464801	Sheep/Goat	Norway	2007	29	Absent	Present	EnteroBase	ERR10464801
ERR10464793	Sheep/Goat	Norway	2015	29	Absent	Present	EnteroBase	ERR10464793

SRR5185399	Human	Germany	1996	29	Absent	Present	EnteroBase	SRR5185399
SRR5185402	Human	Germany	1996	29	Absent	Present	EnteroBase	SRR5185402
ERR12102689	Human	Belgium	2023	29	Absent	Present	EnteroBase	ERR12102689
SRR12263124	Human	United Kingdom	2020	29	Present	Absent	EnteroBase	SRR12263124
SRR12263119	Human	United Kingdom	2020	29	Absent	Present	EnteroBase	SRR12263119
SRR12323710	Human	United Kingdom	2020	29	Present	Absent	EnteroBase	SRR12323710
SRR12323713	Human	United Kingdom	2020	29	Absent	Present	EnteroBase	SRR12323713
ERR12186885	Human	Germany	2020	29	Absent	Present	EnteroBase	ERR12186885
ERR12186914	Human	Germany	2022	29	Absent	Present	EnteroBase	ERR12186914
ERR12186899	Human	Germany	2021	29	Absent	Present	EnteroBase	ERR12186899
ERR12186909	Human	Germany	2022	29	Absent	Present	EnteroBase	ERR12186909
ERR12186892	Human	Germany	2020	29	Absent	Present	EnteroBase	ERR12186892
SRR12798022	Human	United Kingdom	2020	29	Present	Absent	EnteroBase	SRR12798022
SRR12798026	Human	United Kingdom	2020	29	Absent	Absent	EnteroBase	SRR12798026
SRR28232600	Human	United Kingdom	2024	29	Absent	Present	EnteroBase	SRR28232600
SRR28281786	Human	United Kingdom	2024	29	Absent	Present	EnteroBase	SRR28281786
SRR28428297	Human	United Kingdom	2024	29	Absent	Present	EnteroBase	SRR28428297
SRR28428296	Human	United Kingdom	2024	29	Absent	Present	EnteroBase	SRR28428296
ERR13451038	Human	Belgium	2024	29	Absent	Present	EnteroBase	ERR13451038
SRR28674184	Human	United Kingdom	2024	29	Present	Absent	EnteroBase	SRR28674184
SRR28740470	Human	United Kingdom	2024	29	Absent	Present	EnteroBase	SRR28740470
SRR28740451	Human	United Kingdom	2024	29	Absent	Present	EnteroBase	SRR28740451
SRR29046114	Human	United Kingdom	2024	29	Absent	Present	EnteroBase	SRR29046114
SRR29071005	Human	United Kingdom	2023	29	Absent	Absent	EnteroBase	SRR29071005
SRR29091287	Human	United Kingdom	2023	29	Absent	Absent	EnteroBase	SRR29091287
SRR6015209	Human	United States	2015	29	Absent	Present	EnteroBase	SRR6015209
SRR6051780	Human	United Kingdom	2015	29	Absent	Present	EnteroBase	SRR6051780
SRR29183979	Human	United Kingdom	2023	29	Absent	Present	EnteroBase	SRR29183979
SRR29200377	Human	United Kingdom	2023	29	Absent	Present	EnteroBase	SRR29200377
SRR29208712	Human	United Kingdom	2024	29	Absent	Absent	EnteroBase	SRR29208712
SRR29225608	Human	United Kingdom	2023	29	Absent	Absent	EnteroBase	SRR29225608
SRR29252410	Human	United Kingdom	2023	29	Absent	Absent	EnteroBase	SRR29252410
SRR29548348	Human	United Kingdom	2024	29	Absent	Absent	EnteroBase	SRR29548348
SRR29759269	Human	United Kingdom	2024	29	Absent	Absent	EnteroBase	SRR29759269

ERR14085390	Human	France	2024	29	Absent	Present	EnteroBase	ERR14085390
SRR30325407	Human	Denmark	2022	29	Absent	Present	EnteroBase	SRR30325407
SRR30325417	Human	Denmark	2023	29	Absent	Present	EnteroBase	SRR30325417
SRR30325622	Human	Denmark	2023	29	Absent	Present	EnteroBase	SRR30325622
SRR7251001	Human	United Kingdom	2017	29	Present	Absent	EnteroBase	SRR7251001
SRR30610689	Human	United Kingdom	2024	29	Absent	Absent	EnteroBase	SRR30610689
ERR14085475	Human	Belgium	2024	29	Absent	Present	EnteroBase	ERR14085475
SRR31129137	Human	United Kingdom	2024	29	Absent	Present	EnteroBase	SRR31129137
SRR31176584	Human	United Kingdom	2024	29	Absent	Present	EnteroBase	SRR31176584
SRR31369203	Human	United Kingdom	2024	29	Absent	Present	EnteroBase	SRR31369203
SRR31369191	Human	United Kingdom	2024	29	Absent	Present	EnteroBase	SRR31369191
SRR31633555	Human	United States	2024	29	Absent	Present	EnteroBase	SRR31633555
SRR31893753	Human	United Kingdom	2024	29	Present	Absent	EnteroBase	SRR31893753
SRR32044638	Human	United Kingdom	2025	29	Absent	Present	EnteroBase	SRR32044638
SRR32174177	Human	United Kingdom	2025	29	Absent	Present	EnteroBase	SRR32174177
SRR8767901	Human	Austria	2016	29	Absent	Absent	EnteroBase	SRR8767901
SRR14482311	Human	United Kingdom	2021	29	Absent	Present	EnteroBase	SRR14482311
ERR14767991	Human	Belgium	2025	29	Absent	Present	EnteroBase	ERR14767991
SRR15043703	Human	United Kingdom	2021	29	Absent	Present	EnteroBase	SRR15043703
SRR15096260	Human	United Kingdom	2021	29	Absent	Present	EnteroBase	SRR15096260
SRR15096265	Human	United Kingdom	2021	29	Absent	Present	EnteroBase	SRR15096265
SRR15312436	Human	United Kingdom	2021	29	Absent	Present	EnteroBase	SRR15312436
SRR15312438	Human	United Kingdom	2021	29	Absent	Present	EnteroBase	SRR15312438
SRR15361668	Human	United Kingdom	2019	29	Absent	Absent	EnteroBase	SRR15361668
SRR15299522	Human	United Kingdom	2021	29	Absent	Present	EnteroBase	SRR15299522
SRR15299530	Human	United Kingdom	2021	29	Absent	Present	EnteroBase	SRR15299530
SRR15299551	Human	United Kingdom	2021	29	Absent	Present	EnteroBase	SRR15299551
V0147	Cattle	United Kingdom	2018	21	Present	Present	This study	SRR31141847
V0322	Cattle	United Kingdom	2019	21	Present	Present	This study	SRR31141846
V0323	Cattle	United Kingdom	2019	21	Present	Present	This study	SRR31141835
V0324	Cattle	United Kingdom	2019	21	Present	Present	This study	SRR31141833
V0326	Cattle	United Kingdom	2019	21	Present	Present	This study	SRR31141832
V0328	Cattle	United Kingdom	2019	21	Present	Present	This study	SRR31141831

V0338	Cattle	United Kingdom	2019	21	Present	Present	This study	SRR31141830
V0339	Cattle	United Kingdom	2019	21	Present	Present	This study	SRR31141829
V0340	Cattle	United Kingdom	2019	29	Absent	Absent	This study	SRR31141828
V0342	Cattle	United Kingdom	2019	21	Present	Present	This study	SRR31141827
V0343	Cattle	United Kingdom	2019	21	Present	Present	This study	SRR31141845
V0344	Cattle	United Kingdom	2019	21	Present	Present	This study	SRR31141844
V0345	Cattle	United Kingdom	2019	29	Absent	Absent	This study	SRR31141843
V0346	Cattle	United Kingdom	2019	21	Present	Present	This study	SRR31141842
V0347	Cattle	United Kingdom	2019	101	Present	Present	This study	SRR31141841
V0348	Cattle	United Kingdom	2019	29	Absent	Absent	This study	SRR31141840
V0349	Cattle	United Kingdom	2019	29	Absent	Absent	This study	SRR31141839
V0350	Cattle	United Kingdom	2019	29	Absent	Absent	This study	SRR31141838
V0352	Cattle	United Kingdom	2019	29	Absent	Absent	This study	SRR31141837
V0353	Cattle	United Kingdom	2019	29	Absent	Absent	This study	SRR31141836
V0354	Cattle	United Kingdom	2019	29	Absent	Absent	This study	SRR31511900
V0355	Cattle	United Kingdom	2019	297	Absent	Absent	This study	SRR31141834
NC_013361	Human	Japan	2001	21	Present	Absent	NCBI	NC_013361

Supplementary Table S4. Average single nucleotide polymorphism distances of study *Escherichia coli* O26 sequence types 21 and 29 across farm visits.

	Visit	Average SNP Distance	SD	Median
**ST21**	1	NA	NA	NA
	2	9	6	7
	3	14	6	15
	4	NA	NA	NA
	1 & 2	10	2	9
	1 & 3	9	4	10
	1 & 4	12	NA	NA
	2 & 3	17	6	18
	2 & 4	21	4	22
	3 & 4	12	7	10
	1,2,3 & 4	14	6	15
**ST29**	3	12	NA	NA
	4	4	2	4
	3 & 4	8	4	8

SNP = single nucleotide polymorphism, SD = standard deviation, ST = sequence type.

Supplementary Table S5. Chi Square Test Statistics comparing STEC positivity, association with location, sample type, and season

Variables	STEC O26 +ve	STEC O26 -ve	Total	Chi Square Test Statistics
Indoor sample	6	101	107	X^2 = 0.0000198<br>df = 1<br>P value = 0.996
Outdoor sample	8	135	143	
Total	14	236	250	
Pooled faeces	9	132	141	X^2 = 0.757<br>df = 1<br>P value = 0.384
Individual faeces	2	58	60	
Total	11	190	201	
Youngstock sample	10	124	134	X^2 = 1.721<br>df = 1<br>P value = 0.190
Adult sample	4	108	112	
Total	14	232	246	
Visit 1	1	9	10	X^2 = 0.201<br>df = 1<br>*P* value = 0.654
Visit 2	5	75	80	
Total	6	84	90	
Visit 1	1	9	10	

Visit 3	7	73	80	X^2 = 0.017<br>df = 1<br>*P* value = 0.896
Total	8	82	90	
Visit 1	1	9	10	X^2 = 3.132<br>df = 1<br>*P* value = 0.077
Visit 4	1	79	80	
Total	2	88	90	
Visit 2	5	75	80	X^2 = 0.360<br>df = 1<br>*P* value = 0.548
Visit 3	7	73	80	
Total	12	148	160	
Visit 3	7	73	80	X^2 = 4.737<br>df = 1<br>*P* value = 0.03
Visit 4	1	79	80	
Total	8	152	160	
Visit 2	5	75	80	X^2 = 2.771<br>df = 1<br>P value = 0.096
Visit 4	1	79	80	
Total	6	154	160	

Supplementary Table S6. Sequence type 21 and 29 *E. coli* O26 study isolate pairwise single nucleotide polymorphism distance matrix.

Reference = *E. coli* O26:H11 isolate 11368 (Accession:NC_013361), ST=sequence type, green highlight = ≤10 SNPs, orange highlight = >10 and ≤20 SNPs , red highlight = >20 and ≤30 SNPs

				ST21	ST21	ST21	ST21	ST21	ST21	ST21	ST21	ST29	ST21	ST21	ST21	ST29	ST21	ST29	ST29	ST29	ST29	ST29	ST29
				Visit 1	Visit 2					Visit 3							Visit 4						
			Reference	V0147	V0322	V0323	V0324	V0326	V0328	V0338	V0339	V0340	V0342	V0343	V0344	V0345	V0346	V0348	V0349	V0350	V0352	V0353	V0354
		Reference		1033	1170	1137	1131	1075	1174	1134	974	980	1001	972	997	1042	999	998	1034	978	1017	993	945
ST21	Visit 1	V0147	1033		11	9	8	8	12	3	7	932	14	12	10	946	12	950	951	933	941	947	904
ST21	Visit 2	V0322	1170	11		6	5	8	13	16	16	1036	7	27	18	1054	22	1060	1066	1028	1026	1054	978
ST21		V0323	1137	9	6		3	3	15	13	15	1018	7	22	22	1041	19	1038	1044	1004	1010	1032	954
ST21		V0324	1131	8	5	3		3	18	14	15	1004	8	22	21	1018	18	1028	1030	1001	996	1019	951
ST21		V0326	1075	8	8	3	3		15	14	17	985	19	19	20	1001	23	1004	1005	979	978	999	930
ST21		V0328	1174	12	13	15	18	15		11	19	1047	18	29	21	1067	24	1069	1068	1038	1033	1064	985
ST21	Visit 3	V0338	1134	3	16	13	14	14	11		11	1011	16	18	14	1035	17	1031	1044	1010	1016	1028	964
ST21		V0339	974	7	16	15	15	17	19	11		883	17	4	7	895	4	903	900	880	889	892	853
ST29		V0340	980	932	1036	1018	1004	985	1047	1011	883		913	907	916	12	902	5	2	2	5	4	6
ST21		V0342	1001	14	7	7	8	19	18	16	17	913		23	20	921	21	937	934	904	902	925	856
ST21		V0343	972	12	27	22	22	19	29	18	4	907	23		6	922	10	928	928	909	914	920	878
ST21		V0344	997	10	18	22	21	20	21	14	7	916	20	6		927	8	932	932	914	921	928	887
ST29		V0345	1042	946	1054	1041	1018	1001	1067	1035	895	12	921	922	927		920	10	10	12	11	11	13
ST21	Visit 4	V0346	999	12	22	19	18	23	24	17	4	902	21	10	8	920		920	936	916	924	919	886
ST29		V0348	998	950	1060	1038	1028	1004	1069	1031	903	5	937	928	932	10	920		3	4	5	4	3
ST29		V0349	1034	951	1066	1044	1030	1005	1068	1044	900	2	934	928	932	10	936	3		2	2	2	5
ST29		V0350	978	933	1028	1004	1001	979	1038	1010	880	2	904	909	914	12	916	4	2		3	4	6
ST29		V0352	1017	941	1026	1010	996	978	1033	1016	889	5	902	914	921	11	924	5	2	3		0	5
ST29		V0353	993	947	1054	1032	1019	999	1064	1028	892	4	925	920	928	11	919	4	2	4	0		5
ST29		V0354	945	904	978	954	951	930	985	964	853	6	856	878	887	13	886	3	5	6	5	5	

Supplementary Table S7. Estimated coverage and depth of study isolates raw short-read sequences.

Percentage covered and average mapping depth with respect to reference genome *E. coli* U00096.3 (strain K-12) for the paired-end short reads after trimming low quality call endings.

Isolate ID	Average read length (bp)	Percentage mapped reference genome	Average mapping depth
V0147	140	92.35	67.38
V0322	145	92.11	83.88
V0323	142	92.21	61.24
V0324	137	92.2	55.92
V0326	141	92.24	85.15
V0328	141	92.22	73.14
V0338	136	92.2	50.48
V0339	137	92.33	38.62
V0340	135	92.35	50.74
V0342	134	92.16	28.03
V0343	141	92.16	56.13
V0344	137	92.26	44.19
V0345	138	92.37	62.8
V0346	148	92.24	39.08
V0347	141	92.36	33.22
V0348	148	92.37	60.33
V0349	130	92.38	67.94
V0350	148	92.23	45.44
V0352	147	93.9	66.65
V0353	148	93.36	54.5
V0354	141	92.52	35.56
V0355	145	91.31	33.58

Bayesian logistic regression modelling

A Bayesian logistic regression model was fitted to evaluate the association between sample-level factors or predictors and a binary outcome (STEC presence or absence) using the stan_glm function in R (version 4.4.3) with a binomial family and logit link. The predictors included location (Indoor vs. Outdoor), sample type (pooled faeces, individual faeces, environmental), and Visits (Visit1, Visit2, Visit3, Visit4). The dataset comprised 250 observations, with weakly informative normal priors (mean = 0, scale = 2.5) applied to the intercept and predictor coefficients to regularise estimates. Markov Chain Monte Carlo (MCMC) sampling generated 4,000 posterior draws to estimate parameter distributions. Model convergence was assessed using the potential scale reduction factor (Rhat, target ≈ 1) and effective sample size (n_eff). Posterior predictive checks were performed to evaluate model fit, with the mean posterior predictive distribution (mean_PPD) compared to the observed event rate. Odds ratios (OR), 95% credible intervals (CI), and posterior probabilities of OR > 1 (P(OR > 1)) were computed to interpret predictor effects. All analyses were conducted in a Bayesian framework to account for parameter uncertainty.

The Bayesian logistic regression model estimated the effects of location, sample type, and Visits on the likelihood of the binary outcome, with an observed event rate of 5.6% (14/250), closely matched by the mean_PPD (0.0586, SD = 0.0202, 80% CI: 0.032–0.084), indicating good model fit. Supplementary Table 9 summarises the posterior estimates, odds ratios, credible intervals, and posterior probabilities.

The baseline scenario (Indoor, Pooled faeces, Visit1) had a log-odds of -2.1017 (OR = 0.122), corresponding to a ~10.9% probability. Relative to this baseline, outdoor locations were associated with reduced odds (OR = 0.286, 95% CI: 0.074–1.104, P(OR > 1) = 0.035), suggesting a 96.5% probability of a protective effect. Visit4 also showed a strong reduction in odds (OR = 0.108, 95% CI: 0.006–2.337, P(OR > 1) = 0.074), while Visit3 had the highest estimated increase (OR = 1.993, 95% CI: 0.217–35.551, P(OR > 1) = 0.695), followed by Visit2 (OR = 1.728, 95% CI: 0.176–32.840, P(OR > 1) = 0.639). Sample type effects were less conclusive; pooled faeces samples had the highest odds of the outcome, while individual faeces (OR = 0.451, 95% CI: 0.071–2.083, P(OR > 1) = 0.176) and environmental samples (OR = 0.752, 95% CI: 0.163–3.011, P(OR > 1) = 0.361) showed reduced odds, though wide credible intervals indicate substantial uncertainty.

Model diagnostics confirmed convergence (all Rhat ≤ 1.0049) and sufficient effective sample sizes (n_eff ≥ 1177 for all parameters). Monte Carlo standard errors (mcse) were low (e.g., 0.0357 for Intercept, 0.0003 for mean_PPD), supporting precise posterior estimates. The wide credible intervals for some predictors reflect uncertainty due to the low event rate.

Supplementary Table S10: Bayesian Logistic Regression Results (n = 250)

Predictor	Log-Odds Estimate	Odds Ratio	95% Credible Interval (OR)	P(OR > 1)	Interpretation
**Intercept (Indoor, Pooled, Visit1)**	-2.1017	0.122	(0.007, 0.970)	—	Baseline probability ~10.9%
**Location: Outdoor**	-1.2507	0.286	(0.074, 1.104)	0.035	Likely protective effect
**SampleType: Individual faeces**	-0.7962	0.451	(0.071, 2.083)	0.176	Possible reduction in odds
**SampleType: Environmental**	-0.2853	0.752	(0.163, 3.011)	0.361	Uncertain effect
**Visit2**	0.5468	1.728	(0.176, 32.840)	0.639	Possible increase in odds
**Visit3**	0.6895	1.993	(0.217, 35.551)	0.695	Likely increase in odds
**Visit4**	-2.2247	0.108	(0.006, 2.337)	0.074	Likely protective effect

The Bayesian logistic regression model provides evidence of varying effects of location, sample type, and Visits on the likelihood of the binary outcome of STEC presence or absence. The model suggests reduced odds of STEC detection at outdoor sites and during Visit 4, with high posterior probabilities supporting a protective effect. Visit 2 and Visit 3 showed elevated odds, indicating possible temporal variation in STEC prevalence. Sample type effects with Individual faeces and Environmental samples showing reduced odds compared to Pooled faeces were uncertain due to wide intervals and moderate posterior probabilities suggest these effects are not robustly supported. The low event rate limits statistical power and inflates uncertainty, particularly for Visit-related effects. Despite this, model diagnostics and predictive accuracy support overall model adequacy.

These findings have implications for STEC surveillance strategies. The higher odds during initial visits suggesting temporal changes in the outcome. The protective effect of outdoor locations may reflect lower pathogen persistence in open environments or dilution effect. Pooled faeces samples appear more sensitive for detection, though further data are needed to confirm sample type effects. Future studies should aim to increase sample size or event rates to reduce uncertainty and explore additional predictors. Overall, the results underscore the importance of temporal and spatial considerations in optimising STEC detection efforts.

Supplementary Figure S2. Bayesian Posterior predictive check: observed vs. simulated data.

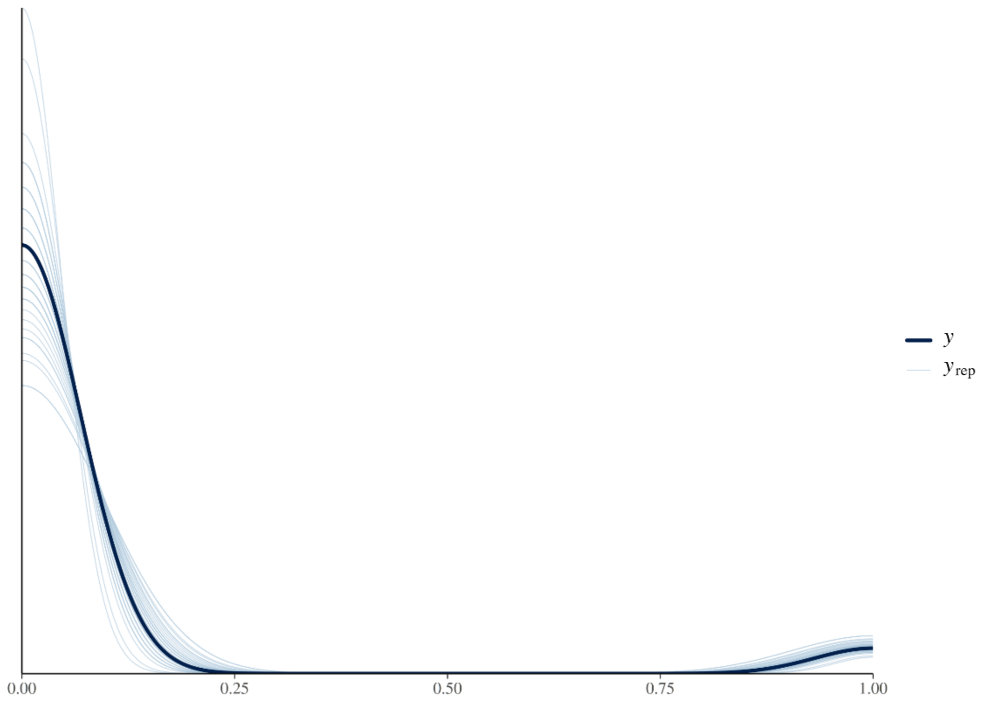

Supplementary Figure S3. Bayesian posterior distribution of odd ratios (95% credible intervals)

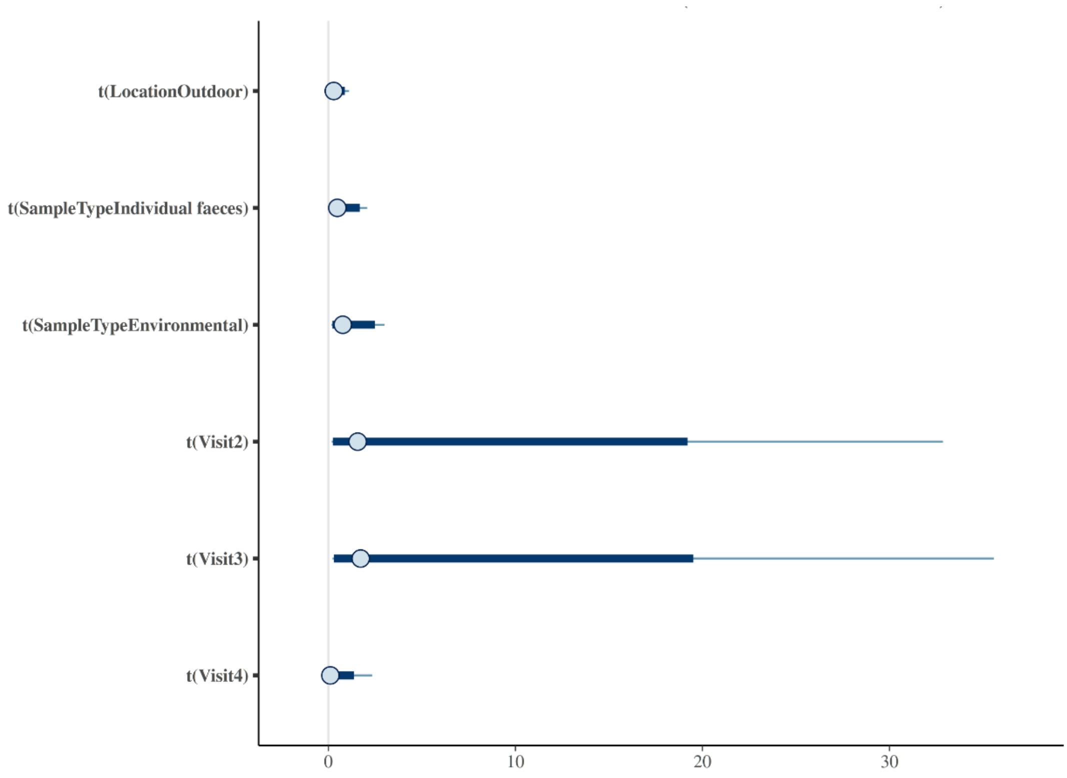

Supplementary Figure S4. Markov Chain Monte Carlo trace plots

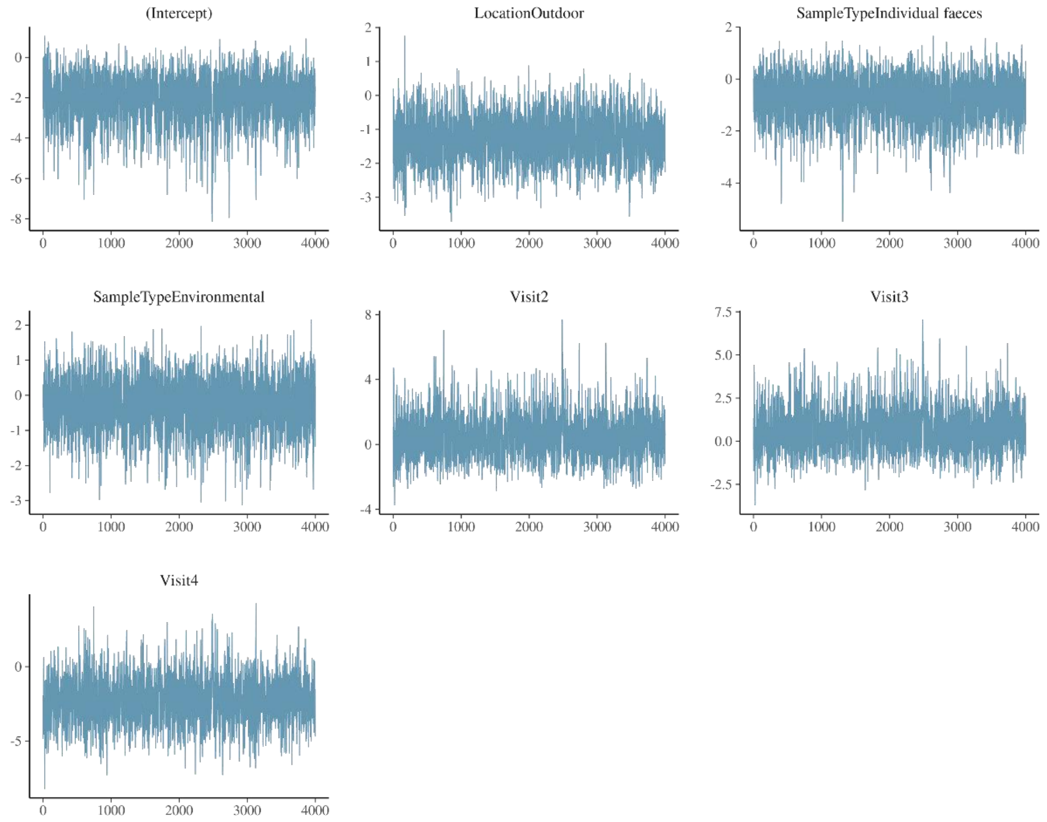

Supplementary Figure S5. Bayesian predicted probabilities by location.

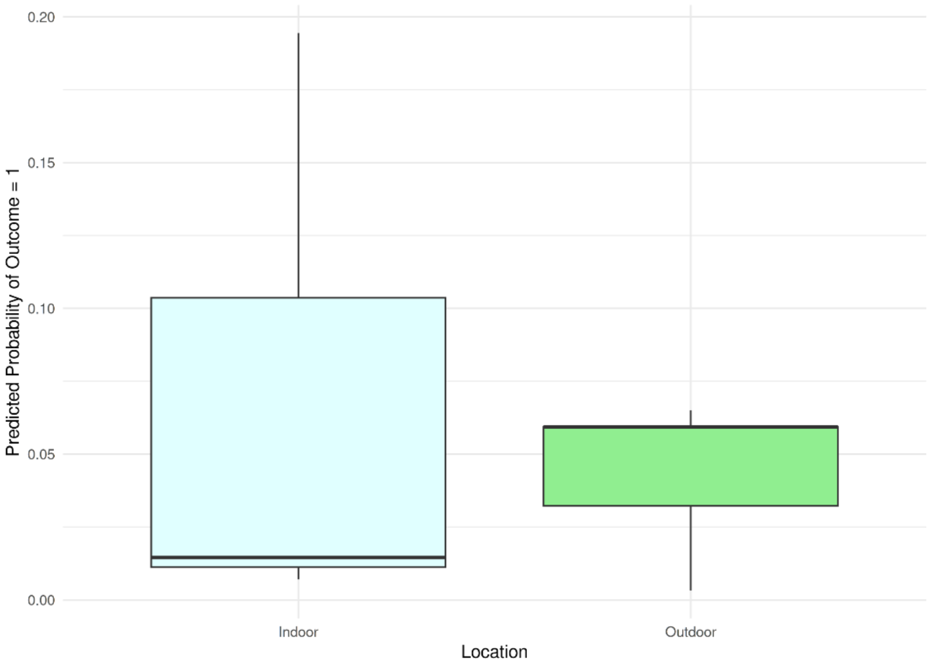

Supplementary Figure S6. Bayesian predicted probabilities by sample type.

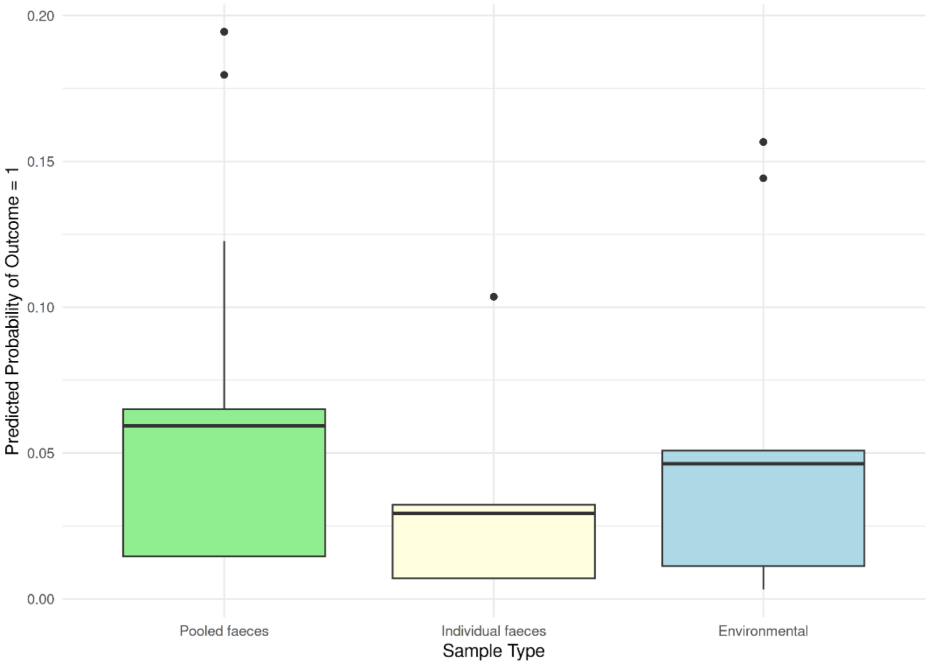

Supplementary Figure S7. Bayesian predicted probabilities by farm visit.

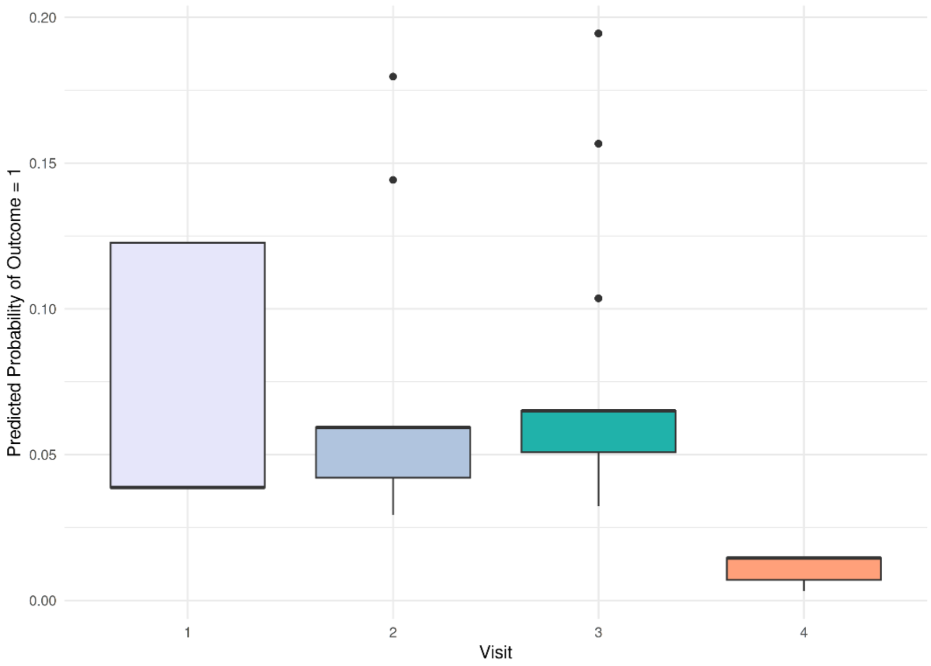
0.20
0.15
0.10
0.05
0.00
Predicted Probability of Outcome = 1
1
2
3
4
Visit
